# ScanITD: Detecting internal tandem duplication with robust variant allele frequency estimation

**DOI:** 10.1093/gigascience/giaa089

**Published:** 2020-08-27

**Authors:** Ting-You Wang, Rendong Yang

**Affiliations:** The Hormel Institute, University of Minnesota, 801 16th Ave NE, Austin, MN 55912, USA; The Hormel Institute, University of Minnesota, 801 16th Ave NE, Austin, MN 55912, USA; Masonic Cancer Center, University of Minnesota, 425 E. River Pkwy, Minneapolis, MN 55455, USA

**Keywords:** internal tandem duplications, FLT3, acute myeloid leukemia, TCGA, chimeric alignment, variant allele frequency

## Abstract

**Background:**

Internal tandem duplications (ITDs) are tandem duplications within coding exons and are important prognostic markers and drug targets for acute myeloid leukemia (AML). Next-generation sequencing has enabled the discovery of ITD at single-nucleotide resolution. ITD allele frequency is used in the risk stratification of patients with AML; higher ITD allele frequency is associated with poorer clinical outcomes. However, the ITD allele frequency data are often unavailable to treating physicians and the detection of ITDs with accurate variant allele frequency (VAF) estimation remains challenging for short-read sequencing.

**Results:**

Here we present the ScanITD approach, which performs a stepwise seed-and-realignment procedure for ITD detection with accurate VAF prediction. The evaluations on simulated and real data demonstrate that ScanITD outperforms 3 state-of-the-art ITD detectors, especially for VAF estimation. Importantly, ScanITD yields better accuracy than general-purpose structural variation callers for predicting ITD size range duplications.

**Conclusions:**

ScanITD enables the accurate identification of ITDs with robust VAF estimation. ScanITD is written in Python and is open-source software that is freely accessible at https://github.com/ylab-hi/ScanITD.

## Findings

### Background

Internal tandem duplication (ITD) is a tandem duplication event residing within coding exons. ITD is a type of genetic alterations that frequently occur in genes implicated in cancer [1]. For example, ITDs in *FLT3* are discovered in ~20%–30% of patients with acute myeloid leukemia (AML) and have been associated with increased relapse risk and decreased overall survival [2, 3]. The *FLT3* ITDs vary in size from 3 to >300 bp and consist of tandem repeats of the entire or partial *FLT3* exon 13–15 region inserted into the FLT3 juxtamembrane domain or nearby tyrosine kinase domain [4, 5]. *FLT3* ITD allele frequency is used in the risk stratification of *FLT3* ITD–positive AML patients; patients with a high allele frequency (>0.5) belong to the high-risk group according to European LeukemiaNet guidelines [6]. *FLT3* ITD with a high allele frequency confers a poor prognosis and has a significant negative effect on the management of patients with AML [6, 7].

The recent development of next-generation sequencing (NGS) has enabled the detection of ITDs at single-nucleotide resolution. However, the detection of larger *FLT3* ITDs and accurate reporting of ITD frequency remains challenging for NGS-based methods. False-negative ITD results or inaccurate variant allele frequency (VAF) estimations could negatively alter treatment solutions for patients with AML. Small and intermediate-sized ITDs can be detected by existing insertion and deletion (indel) callers (e.g., Pindel [8]), and large ITDs are generally identified by tools designed for structural variation (SV) detection. To date, there is a lack of tools specifically designed for ITD detections across the whole size spectrum and accurately reporting the VAF.

In this study, we developed a novel computational tool named ScanITD, which uses chimeric alignments to reconstruct ITDs spanning several tens to several hundreds of base pairs and then performs local realignment of clustered split reads to estimate the VAF of predicted ITDs accurately. Here, we compared the performance of ScanITD with existing ITD detectors and SV detectors using simulated data. We also applied ScanITD to the 50× whole-genome sequencing (WGS) data of NA12878 human individual and whole-exome sequencing (WES) data of 24 samples from patients with AML from The Cancer Genome Atlas (TCGA) project. We demonstrated that ScanITD outperformed the existing methods for detecting ITDs and estimating VAF with high accuracy.

## Methods

### The overall workflow of ScanITD

**Figure 1:**
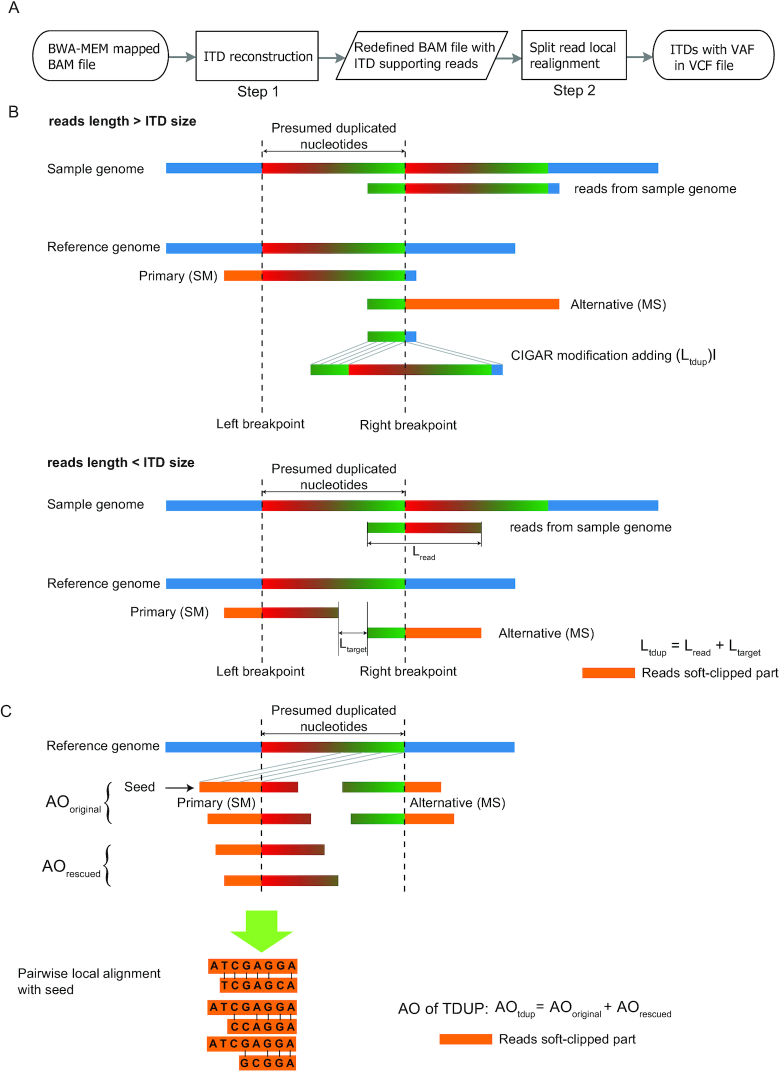
ITD detection with ScanITD. (A) Schematic overview of ScanITD; (B) ITDs are recovered from primary (soft-clipping mode SM) and alternative alignments (soft-clipping mode MS) of chimeric reads (ITD size ≥ read length); C) Split read local realignment to calculate the actual number of ITD supporting reads (labeled AO) that is the sum of ITD-containing seed read and split reads clipped at the same genomic location with seed reads.

The short reads are aligned first by BWA-MEM [9] or other soft-clipping aware NGS aligners to a BAM file, and then ScanITD analyzes the BAM file to detect ITDs following 2 steps (Fig. 1A). In the first step, ScanITD reconstructs ITDs by redefining chimeric reads through the following procedures:

1. Identifying the soft-clipping mode of primary and alternative alignments from the chimeric reads based on their Compact Idiosyncratic Gapped Alignment Record (CIGAR) strings. The primary and alternative alignments due to an ITD event will have different soft-clipping modes, such as left part mapped and right part soft-clipped (referred as MS mode) or left part soft-clipped and right part mapped (referred as SM mode) (Fig. 1B).
2. The primary and alternative alignments must be mapped in the same chromosome and the same strand.
3. The genomic location and size of the ITD are determined from the primary alignment and the distance offset between primary and alternative alignments under 2 scenarios:
(a) If ITD size is less than the read length, ScanITD reconstructs ITDs as insertions based on the transIndel algorithm [10], which will modify the CIGAR string and update the start position of the chimeric read (Fig. 1B). ScanITD will add (n)I in the redefined CIGAR string, where (n) is the size of the ITD and “I” denotes the insertion. A string rotation algorithm as described in Algorithm 1 and Supplementary Fig. S1 will be executed to further evaluate whether the detected event is a novel sequence insertion or a bona fide ITD event.
(b) If the ITD size is larger than the read length, ScanITD will add a new SV tag in the chimeric reads instead of modifying their CIGAR strings. The format of the SV tag follows (TDUP, POS, SIZE), where TDUP indicates that this is an ITD event, and the position and size of the ITD are inferred as illustrated in Fig. 1B.

In the second step, ScanITD will scan the ITD reconstructed BAM file to calculate the VAF of the predicted ITDs. VAF is calculated by AO/DP, where AO (alternate allele observation count) is the number of ITD-supporting reads and DP is the total read depth. AO is counted from both ITD-containing chimeric reads and split reads clipped at the same genomic location with chimeric reads. As shown in Fig. 1C, the soft-clipped part of the ITD-containing read is used as seed sequence and each mismatched alignment of 3′ or 5′ read ends flanking the ITD will be aligned in pairwise with the seed using the Smith-Waterman algorithm to add them in support of the ITD. With this procedure, soft-clipped reads resulting from ITD that were not recognized as ITD-containing reads in Step 1.3.b will be rescued in AO estimation to accurately measure the ITD allele frequency. Finally, the predicted ITDs will be reported in VCF format.

### String rotation algorithm to determine duplications from reads with insertions

For the redefined CIGAR string with an inserted sequence in between 2 mapped parts, we used a string rotation method to judge whether the inserted sequence is a duplicated genomic sequence or a novel sequence. The duplication event is inferred on the basis of genomic sequence surrounding the insertion as described below and in Supplementary Fig. S1.

**Table:** 

**Algorithm 1** duplication inference from an inserted sequence
**Require:** Seq_INS_, Len_INS_, Seq_L_, Seq_R_
Seq_INS_— Inserted sequence
Len_INS_ —The length of the inserted sequence
Seq_L_, Seq_R_—The left-side and the right-side genomic sequences adjacent to the inserted sequence from the reference genome; their length is 1 bp less than the inserted sequence.
**insertionInspector**(Seq_INS_, Len_INS_, Seq_L_, Seq_R_)
1. **for***i* = 1 **to** Len_INS_/2 **do**/* left rotation */
2. Seq_INS_ ← Len_INS_^th^ element of Seq_INS_ + first Len_INS_ −1 elements of Seq_INS_
3. Seq_EXTRACT_ ← last *i* element of Seq_L_ + first Len_INS_*- i* elements of Seq_R_
4. ** if countMismatches**(Seq_INS_, Seq_EXTRACT_) < cutoff **then**
5. ** return** true
6. **end if**
7. **end for**
8. **for***i* = 1 **to** Len_INS_/2 **do**/* right rotation */
9. Seq_INS_ ← first Len_INS_ -1 elements of Seq_INS_ + 1^th^ element of Seq_INS_
10. Seq_EXTRACT_ ← last Len_INS_*- i* elements of Seq_L_ + first *i* elements of Seq_R_
11. **if countMismatches**(Seq_INS_, Seq_EXTRACT_) < cutoff **then**
12. **return** true
13. **end if**
14. **end for**
15. **return** false
**end**

### Simulated ITD dataset for FLT3 region

The simulated data were generated by ITDsim [11], targeting the *FLT3* ITD hotspot region chr13:28,607,161–28,609,590 (hg19). The dataset included a total of 40,401 samples with combinations of varied ITD lengths (range: 1–201 bp; n  = 201) and varied starting positions (chr13:28,608,112–28,608,312; n  = 201). ITD allele frequency was defined as 50% with the mixture of 1,000 paired-end ITD reads and 1,000 paired-end wild-type reads of varied read length (2 × 100 bp and 2 × 275 bp).

### Simulated genome-wide ITD dataset

To evaluate ScanITD and compare it with other widely used duplication detection methods, we rearranged human chromosome 20 (GRCh37/hg19) using the program RSVSim [12] and svsim [13]. In total, we simulated 1,000 tandem duplications with the size ranging from 3 to 300 bp following a β-distribution to reflect the typical ITD size range [5] and real variant size distribution based on an estimate from the Database of Genomic Variants (DGV) [14]. Because ITDs reside within coding exons, we restrict the simulation to coding regions according to the UCSC Genome Browser RefSeq track file.

Based on the rearranged genome and unarranged genome, dwgsim [15] was used to generate synthetic sequence data for use as tumor samples. We generated 36 sets of paired-end reads with varying properties: a mean insert size of 500 bp with 50 bp standard deviation and 75, 100, 150, and 200 bp read lengths at 20×, 50×, and 100× sequence depth each with 10%, 20%, and 50% VAF. A detailed description of the simulation procedure and coding scripts are included in the Supplementary Methods.

### Evaluation metrics for duplication calls

For the simulated ITD dataset against the *FLT3* gene region, we considered the predicted duplication (DUP) calls for each evaluated algorithm to be true-positive (TP) predictions if they met the following criteria: (i) the predicted left breakpoint was within the *FLT3* ITD hotspot region and (ii) the predicted size was equal to the true size. For the simulated genome-wide ITD dataset, we used stringent criteria for the TP definition: (i) the predicted left breakpoint was within 1 bp of the true breakpoint and (ii) the predicted size wass equal to the true size. ITDs could also be detected as short insertions by some algorithms; we counted them as predicted DUP calls. False-positive (FP) predictions are those not satisfying the criteria. False negative (FN) events are DUP events not identified by the detection algorithm. To assess the performance of each tool, we used precision (or positive predictive value), recall (or sensitivity), and F1 score as evaluation metrics as defined below:

\begin{equation*} \mathrm{precision}\,\, = \frac{{\mathrm{TP}}}{{\mathrm{TP} + \mathrm{FP}}}\,\, \end{equation*}

\begin{equation*} \mathrm{recall}\,\, = \frac{{\mathrm{TP}}}{{\mathrm{TP} + \mathrm{FN}}}\,\, \end{equation*}

\begin{equation*} \mathrm{F}1\,\,\mathrm{score}\,\, = \frac{{2\mathrm{TP}}}{{2\mathrm{TP} + \mathrm{FP} + \mathrm{FN}}}\,\, \end{equation*}

### Reference duplication dataset for NA12878 data

A reference DUP dataset corresponding to NA12878 was generated by combining the DUP data identified from the NA12878 assembly generated with long reads (PacBio and Oxford Nanopore Technologies) using Sniffles [16] and the DUP data identified from the NA12878 assembly generated with Illumina short reads using Delly [17], Lumpy [18], and Manta [19]. These datasets are available at [20]. The merge of the DUP datasets was conducted using SURVIVOR [21], after the selection of DUP length $\ge $ 50 bp, resulting in a total of 1,560 DUPs.

### Algorithm evaluation for NA12878 data

NA12878 WGS raw fastq files were obtained from the European Nucleotide Archive (accession No. ERR194147). Paired-end reads were aligned to the GRCh37 human reference using BWA-MEM v0.7.12 with default parameters and duplicated reads were discarded using Picard MarkDuplicates v1.68 [22]. Pindel (v0.2.5) [8], SoftSV (v1.4.2) [23], SvABA (v1.1.3) [24], ScanITD, and Whamg (v1.7.0) [25] were used for DUP calling for NA12878. We excluded ITDSeek; Genomon-ITDetector, which did not work in our computational environment; and Delly, which was used to generate the reference DUP call set.

To reduce confounding effects of detection strategies and differing conventions implemented by the different SV algorithms, we allowed some differences between breakpoint locations for different algorithms when comparing overlaps between DUP call sets with the reference one. Up to 20 bp of difference in the left breakpoints is allowed, 90% overlapped with the reference DUP, and the right breakpoint should not exceed 20 bp of the reference DUP. For tools reporting AO, DP, and AF, such as Pindel and ScanITD, AO $\ge $ 3, DP $\ge $ 10, and AF $\ge $ 0.01 were used as the threshold cut-offs. For SoftSV, the number of supporting reads $\ge $ 3 was the cut-off. We extracted the predicted DUPs ($\ge $50 bp) from all tools we used and compared them against the reference DUP call set from NA12878 to measure the precision and recall of each method.

## Results

### Evaluation of ITD detection algorithms using simulated *FLT3* ITD data

Because ITDs most frequently occur in the *FLT3* gene of patients with AML, we first sought to compare ScanITD with 3 existing ITD detectors: ITDseek v1.2 [11], Genomon-ITDetector [26], and Pindel v0.2.5 [8], using 2 simulation datasets of hotspot *FLT3* ITDs at 275 and 100 bp paired-end reads. ITDseek and Genomon-ITDetector are designed for ITD detection. Pindel has also been reported to perform well in *FLT3* ITD detection [2], so it was included in the comparison. We excluded ITD assembler [27] because it did not work in our computational environment and lacked support from its authors. The FASTQ files with synthetic paired-end reads were aligned by BWA-MEM to obtain BAM files. The BAM files with hard-clipped/soft-clipped reads or reads with small insertions were kept. All the evaluating ITD detectors were called using default parameters with minor adjustments and the analyses were based on the BWA-MEM–aligned BAM files that we used.

**Figure 2:**
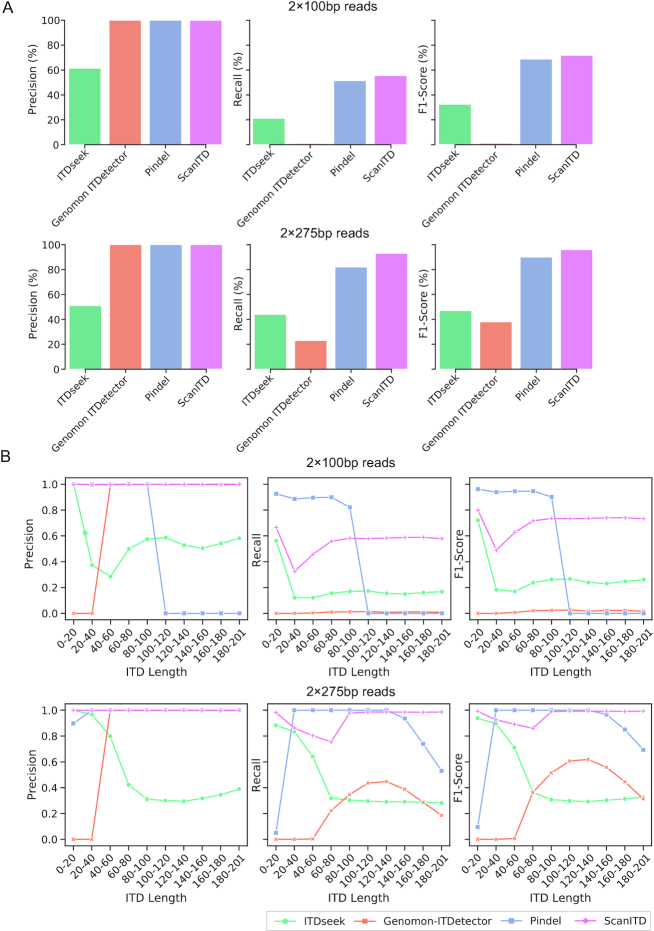
Benchmarking of ScanITD for ITD detection against existing ITD detection tools using 2 × 100 bp and 2 × 275 bp simulated reads. (A) Performance measured by precision (left), recall (middle), and F1 score (right) for ScanITD, ITDseek, Genomon-ITDetector, and Pindel. (B) Performance measured by precision (left), recall (middle), and F1 score (right) at ITD length range from 1 to 201 bp.

We observed that ScanITD achieved the highest recall, precision, and F1 score under these 2 different read length scenarios (Fig. 2A). When further evaluating the recall and precision in a different ITD size range, we found ScanITD to be superior at detecting medium to large size ITDs (>100 bp) compared with other methods (Fig. 2B).

### Evaluation of ITD detection algorithms using simulated genome-wide ITD dataset

To evaluate the performance of ITD detection algorithms in general, we generated a genome-wide ITD simulation dataset allowing unbiased estimation of the sensitivity and specificity of different ITD detection algorithms in other gene regions. To keep a reasonable runtime, we rearranged the target genome sequence based on human chromosome 20, which accounts for 2% of the human genome but has reasonably representative genomic features such as GC content, gene density, and repeat content, compared with the whole genome. Then, we randomly placed 1,000 tandem duplications throughout the target genome. The size of the duplications ranged from 3 to 300 bp. To assess the impact of sequencing properties (i.e., read depth and read length) and duplication properties (i.e., VAF), we generated 36 sets of synthetic paired-end reads with varied read depth, read length, and VAF for the tandem duplications. Last, the simulation data were aligned to the human reference genome (GRCh37/hg19) using BWA-MEM.

Algorithms that were developed for general use to identify SVs could detect tandem duplications at a larger scale. Therefore, we expanded our comparison to include several widely used SV detection tools. Besides the 3 ITD detectors (ITDseek, Genomon-ITDetector, and Pindel), we compared ScanITD with 4 SV detectors including Delly v0.8.2, SvABA v1.1.3, SoftSV v1.4.2, and Whamg v1.7.0 on the simulated genome-wide ITD datasets with various read depth, read length, and VAF settings. The measurement metrics precision (or positive predictive value), recall (or sensitivity), and F1 score (an overall measure of accuracy that combines precision and recall) were used to assess the performance of different algorithms in the comparison.

**Figure 3:**
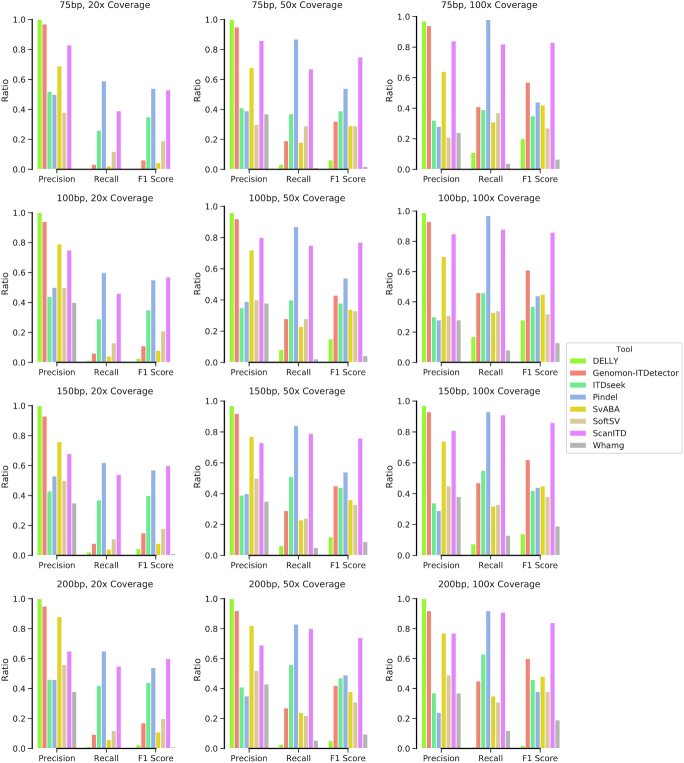
Benchmarking of ScanITD for ITD detection against existing ITD detection tools and SV detection tools using genome-wide simulated reads with 10% VAF.

At the 10% VAF setting, we observed that Delly and Genomon-ITDetector achieved the highest precision across all coverage levels and read lengths, with a poor performance in recall (Fig. 3). ScanITD got the second runner-up position when read length was 75 bp or sequencing depth was 100×, suggesting that it reliably detected tandem duplications in the short-read scenario, especially for the targeted sequencing setting. In terms of sensitivity, Pindel had the highest recall at a cost of low precision, followed by ScanITD in all tested situations at the 10% VAF setting. The differences were negligible at 50/100× coverage with 150/200 bp read lengths. While at 20% and 50% VAF settings, ScanITD showed the same or higher recall compared to Pindel at 50/100× coverage with 150/200 bp read length (Supplementary Figs S2 and S3). When considering both precision and recall, ScanITD achieved the highest F1 score of all the methods tested in all tested situations (Fig. 3, Supplementary Figs S2 and S3), indicating that it could correctly identify real tandem duplications without being disturbed by false-negative results. In general, our results showed that ScanITD had the best overall performance measured by F1 score in detecting tandem duplication events across all conditions.

**Figure 4:**
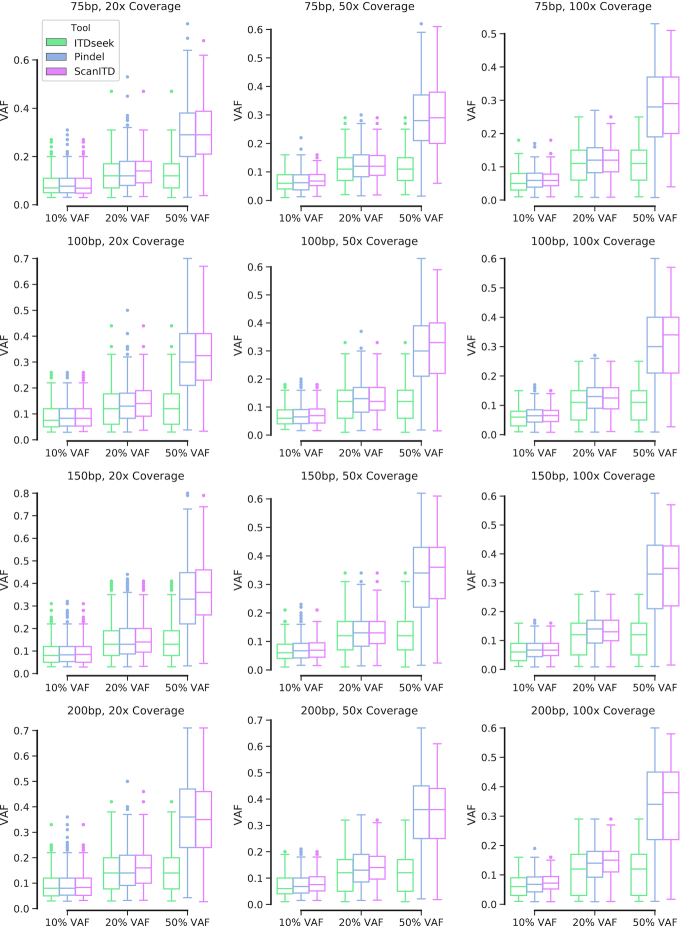
Benchmarking VAF of ScanITD for ITD detection against ITDseek and Pindel using genome-wide simulated reads. The box represents the VAF values between the 1st and 3rd quartiles-the InterQuartile Range(IQR=Q3-Q1), the line across the box indicates the median, the whiskers are lines extending from Q1 and Q3 to endpoins within Q1-1.5×IQR and Q3+1.5×IQR, respectively. Outliers are those that are outside whiskers range.

We next sought to evaluate the performance of the VAF estimation by ScanITD together with ITDseek and Pindel using our simulated data. We chose these 2 existing ITD detectors for comparison because they are the only ITD detectors with the feature of reporting the VAFs of their predicted ITDs. As shown in Fig. 4, all 3 methods tended to have a lower estimation of VAFs comparing their ground truth values, which may be explained by the non-uniform distribution of read coverage [28]. Among them, ScanITD and Pindel reported more accurate VAFs than ITDseek. In general, ScanITD outperformed Pindel with a relatively higher median VAF estimation at most of the VAF settings in either low- or high-coverage datasets.

### Evaluation with NA12878 WGS data

To assess the performance of ScanITD with real DNA sequencing data, we analyzed the well-studied HapMap sample NA12878/HG001. The 100-bp paired-end WGS data with an average coverage of 50× were provided through Illumina's Platinum genomes project [29]. We constructed a reference call set for sample NA12878 by combining duplication events identified from long reads (PacBio and Oxford Nanopore Technologies) and Illumina short reads. Most all of the called duplications are longer than 50 bp, so DUPs with length $\ge $ 50 bp were used in the reference call set.

**Figure 5:**
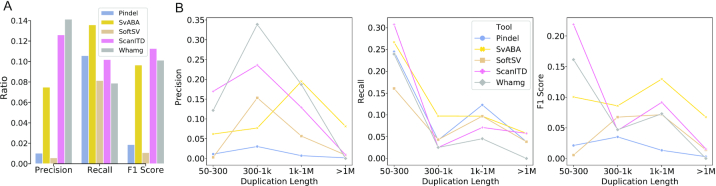
Benchmarking of duplication detection using NA12878 whole-genome sequencing data. (A) Overall performance comparison. (B) Performance comparison in different duplication size range. Precision, recall, and F1 score are used as the performance measurements.

Because ITDseek and Genomon-ITDetector were not capable of detecting any duplications in this NA12878 dataset, we tested ScanITD along with Pindel, SvABA, SoftSV, and Whamg against a reference duplication call set by measuring their precision, recall, and F1 scores. We observed that ScanITD achieved the highest F1 score and second-highest precision/sensitivity among these 5 algorithms (Fig. 5A), suggesting an overall better accuracy of detecting the duplication events in NA12878. When further evaluating the performance in different duplication size ranges, we found that ScanITD was superior at detecting small to medium-size duplications (50–300 bp) compared with other methods (Fig. 5B). Our results indicated that ScanITD is the best approach for detecting ITD range duplication events (≤300 bp).

Finally, we recorded the computational runtime and memory consumption of ScanITD when analyzing the NA12878 WGS dataset using a server equipped with a 16-core Intel Xeon(R) CPU E5–2620 v4 at 2.10 GHz with 16 GB of memory. The first step (ITD reconstruction) took 7 hours and the second step (split read realignment and ITD calling) took 28 hours when running on chromosomes in parallel. Notably, ScanITD is memory-efficient, only requiring 2.2 GB peak memory usage.

### Application to whole-exome data of patients with AML

To examine whether ScanITD could enhance ITD detection in clinical data, we analyzed the WES data from the TCGA AML cohort. It has been reported that 24 patients harbor experimentally validated *FLT3* ITDs [1]. We applied ScanITD together with the 3 existing ITD detectors to identify *FLT3* ITDs from these 24 patients. We used the original reported ITD size (ranging from 18 to 102 bp) as the gold standard [1] to measure the correctness of ITD prediction for each tool. As shown in Table 1, ScanITD correctly identified 22 ITDs and it demonstrated the highest sensitivity (92%) compared with Pindel (72%), ITDseek (42%), and Genomon ITDetector (71%). We further evaluated the reported VAFs for ScanITD, Pindel, and ITDseek that are capable of predicting ITD allele fraction. We found that ScanITD reported the highest VAFs in 20 samples while Pindel only reported the highest VAFs in 4 samples, and ITDseek always reported lower VAFs than ScanITD and Pindel. Thanks to split read local realignment, ScanITD could rescue ITD-supporting reads in the AO calculation, increasing the reported VAF. For example, ScanITD reported an 87-bp *FLT3* ITD with its VAF of 0.30 that is nearly twice the VAF reported by Pindel (0.16) in patient TCGA-AB-2844. A manual review of the aligned reads in this patient further confirmed that ScanITD's estimation of this VAF was accurate (Supplementary Fig. S4). Our results suggest that ScanITD outperforms the existing methods for accurately measuring the VAFs of the ITD predictions.

**Table 1:** *FLT3* ITD detection in TCGA AML samples

		ScanITD		Pindel		ITDseek		Genomon ITDetector (length)
TCGA sample ID	ITD (length)	VAF	Length	VAF	Length	VAF	Length	
TCGA-AB-2812	51	**0.34**	51	0.14	51	0.11	51	51
TCGA-AB-2825	102	0.06	102	**0.17**	102	0.11	97	Missed
TCGA-AB-2830	69	0.01	69	**0.027**	69	0.03	56	42
TCGA-AB-2836	33	**0.08**	33	0.047	33	0.03	34	Missed
TCGA-AB-2840	18	**0.25**	18	0.23	18	0.01	18	18
TCGA-AB-2844	87	**0.30**	87	0.16	87	0.05	89	Missed
TCGA-AB-2853	18	0.21	18	**0.30**	18	0.08	18	18
TCGA-AB-2869	54	**0.21**	54	0.50	46	0.12	55	54
TCGA-AB-2871	63	**0.011**	63	0.0059	63	0.01	63	Missed
TCGA-AB-2875	30	**0.25**	30	0.16	30	0.03	30	30
TCGA-AB-2877	18	**0.22**	18	0.20	18	0.02	18	18
TCGA-AB-2879	33	**0.32**	33	0.20	33	0.06	34	33
TCGA-AB-2880	21	**0.23**	21	0.18	21	0.11	21	21
TCGA-AB-2895	45	**0.23**	45	0.16	45	0.09	49	45
TCGA-AB-2913	66	**0.14**	66	0.065	66	0.06	68	66
TCGA-AB-2915	51	**0.029**	51	0.13	54	0.03	51	51
TCGA-AB-2918	21	0.1	90	0.11	88	0.05	90	88
TCGA-AB-2921	24	0.15	57	0.09	57	0.06	53	24
TCGA-AB-2922	33	**0.25**	33	0.15	33	0.04	33	33
TCGA-AB-2925	42	**0.21**	42	0.10	42	0.11	45	42
TCGA-AB-2930	42	**0.05**	42	0.029	42	0.02	39	42
TCGA-AB-2931	75	**0.15**	75	0.28	70	0.07	72	Missed
TCGA-AB-2934	57	**0.05**	57	0.043	57	0.05	56	57
TCGA-AB-2942	24	**0.15**	24	0.12	24	0.03	24	24

The correct predictions with the highest VAF are highlighted in boldface; incorrect predictions are highlighted as underlined text.

## Discussion

Herein, we have devised ScanITD, a computational approach allowing the accurate identification of ITDs from DNA sequencing data. ScanITD made good use of chimeric alignments for ITD reconstruction. By performing local realignment of clustered split reads, ScanITD achieved robust VAF estimation. The evaluations on simulated and real data demonstrate that ScanITD outperformed the existing ITD detectors, especially for estimating VAF with high accuracy. Compared with general-purpose SV detectors, ScanITD also exhibited competitive performance and superior accuracy in duplication detection, especially for a range of ITD sizes.

Besides performance improvements compared to other ITD callers, 1 improvement of ScanITD is the ability to distinguish insertions of novel sequence and insertions as a result of the duplicated genome sequence. Most of the ITD callers and general-purpose SV detection methods, such as Pindel [8] and SvABA [24], are not able to differentiate small novel sequence insertions from tandem duplications and report both types of events as insertions. In essence, ScanITD belongs to split-read–based approaches leveraging the split reads that solely mapped around ITD breakpoints. Other split-read–based methods, such as Pindel and SoftSV [23], rely on realigning all the split reads. Another novel feature of ScanITD is that it realigns split reads in a heuristic manner that can use all related split reads without limiting the length of the soft-clipped part. However, Pindel and SoftSV realign split reads to reference genome by requiring the soft-clipped part to be of a reasonable length (e.g., >10 bp). The heuristic algorithm used by ScanITD is a seed-and-realignment procedure. Once the seed chimeric read is found, any split reads clipped at the same genomic location as the seed will be taken into consideration, no matter how short they are (Fig. 1C and Supplementary Fig. S4). This strategy makes ScanITD estimate VAFs with high accuracy (Fig. 4 and Table 1). Our benchmarks using simulated data have demonstrated that ScanITD exhibited competitive or superior performance with algorithms using a split-read realignment strategy (e.g., Pindel, SoftSV) or integrated strategy (e.g., Delly integrating split-read and read-pair information) (Fig. 3).

As a heuristic algorithm, ScanITD uses chimeric reads or reads with small insertions to locate the ITD breakpoints; the generation of these ITD indicators solely relies on soft-clipping–aware NGS aligners such as BWA-MEM. However, under certain circumstances depending on ITD length and read length, aligners may generate only soft-clipped/hard-clipped reads instead of chimeric reads or reads with small insertions. Under these cases, ScanITD is not able to determine correct breakpoints (Fig. 2B).

There are still limitations for ScanITD to detect some types of duplication events. Our benchmarks using NA12878 WGS data demonstrated that ScanITD performed weakly compared with general-purpose SV detection algorithms for large duplications. The existence of duplications carrying indels and dispersed duplication might be 2 possible reasons (Supplementary Fig. S5). In these cases, ScanITD is not able to determine correct breakpoints using chimeric reads. A combination and integration of multiple independent pieces of evidence such as read pair and read depth information may further improve ScanITD's performance for non-tandem duplication event detection.

## Conclusions

We present ScanITD as a robust method for detecting ITDs from NGS data and predicting a precise ITD allele fraction. We demonstrated that ScanITD reliably detects medium-size and large ITDs with synthetic and real data and outperformed the existing methods. ScanITD is capable of detecting ITDs across the full size spectrum with base-pair resolution. We anticipate that ScanITD will enable identification and elucidation of clinically important ITDs that are currently difficult to characterize.

## Availability of Supporting Source Code and Requirements

Project name: ScanITD

Project home page: https://github.com/ylab-hi/ScanITD

Operating system(s): platform independent

Programming language: Python

Other requirements: SAMTools (https://www.htslib.org/)

License: MIT License

Biotools identifier: ScanITD (https://bio.tools/ScanITD)

RRID:SCR_018886

## Availability of Supporting Data and Materials

WES data from the TCGA AML cohort are available at the Genomic Data Commons Data Portal [30] (Project ID: TCGA-LAML; dbGaP study accession No.: phs000178). NA12878 WGS fastq data are available at the European Nucleotide Archive (accession No.: ERR194147). An archival copy of the code and supporting data is available via the *GigaScience* GigaDB database [31].

## Additional Files

Supplementary Methods.

Supplementary Figure S1. A string rotation method to determine whether the inserted sequence is from a duplicated genomic sequence or not.

Supplementary Figure S2. Benchmarking of ScanITD for ITD detection against existing ITD detection tools and SV detection tools using genome-wide simulated reads with 20% VAF.

Supplementary Figure S3. Benchmarking of ScanITD for ITD detection against existing ITD detection tools and SV detection tools using genome-wide simulated reads with 50% VAF.

Supplementary Figure S4. Experimentally validated FLT3 ITD (chr13:28608215-28608301) was identified by ScanITD in TCGA-AB-2844 WES data.

Supplementary Figure S5. Illustration of the non-tandem duplication scenarios with chimeric reads.

giaa089_GIGA-D-20-00166_Original_Submission

giaa089_GIGA-D-20-00166_Revision_1

giaa089_Response_to_Reviewer_Comments_Original_Submission

giaa089_Reviewer_1_Report_Original_Submission Medhat Mahmoud -- 6/21/2020 Reviewed

giaa089_Supplemental_File

## Abbreviations

AO: alternate allele observation count; AML: acute myeloid leukemia; BAM: Binary Alignment Map; bp: base pairs; BWA: Burrows-Wheeler Aligner; CPU: central processing unit; DP: read depth; TDUP: tandem duplication; indel: insertion and deletion; ITD: internal tandem duplication; NGS: next-generation sequencing; SV: structural variations; TCGA: The Cancer Genome Atlas; UCSC: University of California Santa Cruz; VAF: variant allele frequency; WGS: whole-genome sequencing; WES: whole-exome sequencing.

## Competing Interests

The authors declare that they have no competing interests.

## Funding

This work was supported by a Research Starter Grant from PhRMA foundation, a Young Investigator Award from the Prostate Cancer Foundation, and an Idea Development Award from Department of Defense Prostate Cancer Research Program (W81XWH-19-1-0161). The work is also supported by The Eagles Telethon Post Doctoral Fellowship to T.-Y. W.

## Authors' Contributions

T.-Y. W. developed the software, performed data analysis, and wrote the manuscript. R.Y. conceptualized the research idea, supervised the development of the software and data analysis, and reviewed and edited the draft.
