# Supplementary material for: ScanITD: Detecting internal tandem duplication with robust variant allele frequency estimation
Source: Gigascience. 2020 Aug 27;9(8):giaa089. doi: 10.1093/gigascience/giaa089 (PMC7450668; doi:10.1093/gigascience/giaa089)
Supplement: giaa089_GIGA-D-20-00166_Revision_1 [file giaa089_giga-d-20-00166_revision_1.pdf]

GigaScience

ScanITD: detecting internal tandem duplication with robust variant allele frequency estimation

--Manuscript Draft--

**Manuscript Number:**	GIGA-D-20-00166R1
**Full Title:**	ScanITD: detecting internal tandem duplication with robust variant allele frequency estimation
**Article Type:**	Technical Note
**Funding Information:**	Pharmaceutical Research and Manufacturers of America Foundation (Research Starter Grant) — Dr. Rendong Yang<br>Prostate Cancer Foundation (Young Investigator Award) — Dr. Rendong Yang<br>DOD Prostate Cancer Research Program (Idea Development Award) — Dr. Rendong Yang
**Abstract:**	Background<br>Internal tandem duplications (ITDs) are tandem duplications within coding exons and are important prognostic markers and drug targets for acute myeloid leukemia (AML). Next-generation sequencing has enabled the discovery of ITD at single nucleotide resolution. ITD allele frequency is used in the risk stratification of AML patients, higher ITD allele frequency has been proved for its association with poorer clinical outcomes. However, the ITD allele frequency data is often unavailable to treating physicians and the detection of ITDs with accurate variant allele frequency (VAF) estimation remains challenging for short read sequencing.<br>Results<br>Here we present ScanITD approach that performs a stepwise seed-and-realignment procedure for ITD detection with accurate VAF prediction. The evaluations on simulated and real data demonstrate that ScanITD outperforms three state-of-the-art ITD detectors, especially for VAF estimation. Importantly, ScanITD yields the best accuracy than general-purpose structural variations callers for predicting ITD size range duplications.<br>Conclusions<br>ScanITD enables the accurate identification of ITDs with robust VAF estimation. ScanITD is written in Python, and is open-source software and freely accessible at https://github.com/ylab-hi/ScanITD .
**Corresponding Author:**	Rendong Yang<br>University of Minnesota<br>UNITED STATES
**Corresponding Author Secondary Information:**	
**Corresponding Author's Institution:**	University of Minnesota
**Corresponding Author's Secondary Institution:**	
**First Author:**	Ting-You Wang
**First Author Secondary Information:**	
**Order of Authors:**	Ting-You Wang<br>Rendong Yang
**Order of Authors Secondary Information:**	
**Response to Reviewers:**	Dr. Hans Zauner, PhD<br>Assistant Editor of GigaScience<br><br>July 28, 2020<br>RE: GigaScience - GIGA-D-20-00166

Powered by Editorial Manager® and ProduXion Manager® from Aries Systems Corporation

	Dear Dr. Zauner,<br><br>We would like to thank you for accepting manuscript (GIGA-D-20-00166) entitled “ScanITD: detecting internal tandem duplication with robust variant allele frequency estimation” for publication in the journal of GigaScience. We greatly appreciate the critical examination and the constructive comments by the reviewers. The manuscript has been revised for better readability according to the suggestions of the Editor and Reviewers.<br><br>We already have submitted the supporting data to GigaDB (http://dx.doi.org/10.5524/100775). Following Editor’s editorial requests, we added GigaDB citation, provided identifiers of bio.tools and SciCrunch.org, and moved the URLs in the main text to the bibliography. We have made all the necessary corrections.<br><br>We again appreciate the kindness of the Editor and Reviewers in helping improve the manuscript.<br>Thank you again.<br><br>Best regards,<br><br>Rendong Yang, Ph.D.<br>Assistant Professor<br>The Hormel Institute<br>University of Minnesota<br>Tel: 507-437-9660<br>Email: yang4414@umn.edu
**Additional Information:**	
**Question**	**Response**
Are you submitting this manuscript to a special series or article collection?	No
**Experimental design and statistics**<br><br>Full details of the experimental design and statistical methods used should be given in the Methods section, as detailed in our Minimum Standards Reporting Checklist. Information essential to interpreting the data presented should be made available in the figure legends.<br><br>Have you included all the information requested in your manuscript?	No
If not, please give reasons for any omissions below.<br><br>as follow-up to "**Experimental design and statistics**	Not applicable.

Powered by Editorial Manager® and ProduXion Manager® from Aries Systems Corporation

Full details of the experimental design and statistical methods used should be given in the Methods section, as detailed in our Minimum Standards Reporting Checklist. Information essential to interpreting the data presented should be made available in the figure legends.<br><br>Have you included all the information requested in your manuscript?<br><br>"	
**Resources**<br><br>A description of all resources used, including antibodies, cell lines, animals and software tools, with enough information to allow them to be uniquely identified, should be included in the Methods section. Authors are strongly encouraged to cite Research Resource Identifiers (RRIDs) for antibodies, model organisms and tools, where possible.<br><br>Have you included the information requested as detailed in our Minimum Standards Reporting Checklist?	No
If not, please give reasons for any omissions below.<br><br>as follow-up to "**Resources**<br><br>A description of all resources used, including antibodies, cell lines, animals and software tools, with enough information to allow them to be uniquely identified, should be included in the Methods section. Authors are strongly encouraged to cite Research Resource Identifiers (RRIDs) for antibodies, model organisms and tools, where possible.<br><br>Have you included the information	Not applicable.

requested as detailed in our Minimum Standards Reporting Checklist?<br><br>"	
**Availability of data and materials**<br><br>All datasets and code on which the conclusions of the paper rely must be either included in your submission or deposited in publicly available repositories (where available and ethically appropriate), referencing such data using a unique identifier in the references and in the "Availability of Data and Materials" section of your manuscript.<br><br>Have you have met the above requirement as detailed in our Minimum Standards Reporting Checklist?	Yes

Manuscript

Click here to access/download;Manuscript;GIGA-D-20-00166_for_publication.docx

ScanITD: detecting internal tandem duplication with robust variant allele frequency estimation

Ting-You Wang[1] and Rendong Yang[1,2*]

[1]The Hormel Institute, University of Minnesota, Austin MN 55912

[2]Masonic Cancer Center, University of Minnesota, Minneapolis, MN 55455.

*To whom correspondence should be addressed (yang4414@umn.edu).

Ting-You Wang: https://orcid.org/0000-0003-4354-3198

Rendong Yang: https://orcid.org/0000-0002-9512-2240

Abstract

Background
Internal tandem duplications (ITDs) are tandem duplications within coding exons and are important prognostic markers and drug targets for acute myeloid leukemia (AML). Next-generation sequencing has enabled the discovery of ITD at single-nucleotide resolution. ITD allele frequency is used in the risk stratification of AML patients, higher ITD allele frequency has been proved for its association with poorer clinical outcomes. However, the ITD allele frequency data is often unavailable to treating physicians and the detection of ITDs with accurate variant allele frequency (VAF) estimation remains challenging for short read sequencing.

Results
Here we present ScanITD approach that performs a stepwise seed-and-realignment procedure for ITD detection with accurate VAF prediction. The evaluations on simulated and real data demonstrate that ScanITD outperforms three state-of-the-art ITD detectors, especially for VAF estimation. Importantly, ScanITD yields better accuracy than general-purpose structural variations callers for predicting ITD size range duplications.

Conclusions
ScanITD enables the accurate identification of ITDs with robust VAF estimation. ScanITD is written in Python, and is open-source software and freely accessible at https://github.com/ylab-hi/ScanITD.

Keywords

Internal tandem duplications, FLT3, acute myeloid leukemia, TCGA, chimeric alignment, variant allele frequency

Findings

Background

Internal tandem duplication (ITD) is a tandem duplication event reside within coding exons. ITD is a type of genetic alterations that are frequently occurred in genes implicated in cancer [1]. For example, ITDs in *FLT3* are discovered in approximately 20% to 30% of acute myeloid leukemia (AML) patients and have been associated with increased relapse risk and decreased overall survival [2, 3]. The *FLT3*-ITDs are varied in size from 3bp to above 300bp, which are tandem repeats of the entire or partial *FLT3* exon 13 to exon 15 region inserted into the FLT3 juxtamembrane domain or nearby tyrosine kinase domain [4, 5]. *FLT3*-ITD allele frequency is used in the risk stratification of *FLT3*-ITD-positvie AML patients, patients with a high allele frequency (>0.5) belong to the high-risk group according to European LeukemiaNet (ELN) guidelines [6]. *FLT3*-ITD with a high allele frequency confers a poor prognosis and has a significant negative impact on the management of AML patients [6, 7].

The recent development of next-generation sequencing (NGS) has enabled the detection of ITDs at single-nucleotide resolution. However, the detection of larger FLT3-ITD and accurate reporting of ITD frequency remains challenging for NGS based methods. False negative ITD results or inaccurate variant allele frequency (VAF) estimations could negatively alter treatment solutions for AML patients. Small and intermediate sized ITDs can be detected by existing indel callers (e.g. Pindel [8]) and large ITDs are generally identified by tools designed for structural variation (SV) detection. To date, there is a lack of tools specifically designed for ITD detections across the whole size spectrum and accurately report the VAF.

In this study, we developed a novel computational tool named ScanITD, which utilizes chimeric alignments to reconstruct ITDs spanning several tens to several hundreds of base pairs and then performs local realignment of clustered split reads to estimate the VAF of predicted ITDs accurately. Here, we compared the performance of ScanITD with existing ITD detectors and SV detectors using simulated data. We also applied ScanITD to the 50× whole genome sequencing (WGS) data of NA12878 human individual and whole exome sequencing (WES) data of 24 AML patient samples from The Cancer Genome Atlas (TCGA) project. We demonstrated that ScanITD outperformed the existing methods for detecting ITDs and estimating VAF with high accuracy.

Methods

The overall workflow of ScanITD

The short reads are aligned first by BWA-MEM [9] or other soft-clipping aware NGS aligners to a BAM file and then ScanITD analyzes the BAM file to detect ITDs following two steps (**Figure 1A**). In the first step, ScanITD reconstructed ITDs by redefining chimeric reads through the following procedures:

1. Identifying the soft-clipping mode of primary and alternative alignments from the chimeric reads based on their Compact Idiosyncratic Gapped Alignment Record (CIGAR) strings. The primary and alternative alignments due to an ITD event will

have different soft-clipping modes, such as left part mapped and right part soft-clipped (referred as MS mode) or left part soft-clipped and right part mapped (referred as SM mode) (**Figure 1B**).

2. The primary and alternative alignments must be mapped in the same chromosome and the same strand.
3. The genomic location and size of the ITD are determined from the primary alignment and the distance offset between primary and alternative alignments under two scenarios:
    a. If ITD size is less than the read length, ScanITD reconstructed ITDs as insertions based on transIndel algorithm [10] that will modify the CIGAR string and update the start position of the chimeric read (**Figure 1B**). ScanITD will add (n)I in the redefined CIGAR string where (n) is the size of the ITD and 'I' denotes the insertion. A string rotation algorithm as described in **Algorithm 1** and **Supplementary Figure S1** will be executed for further evaluation whether the detected event is a novel sequence insertion or a *bona fide* ITD event.
    b. If the ITD size is larger than the read length, ScanITD will add a new SV tag in the chimeric reads instead of modifying their CIGAR strings. The format of SV tag follows (TDUP, POS, SIZE) where TDUP indicates this is ITD event, and the position and size of the ITD are inferred as illustrated in **Figure 1B**.

In the second step, ScanITD will scan the ITD reconstructed BAM file to calculate the VAF of the predicted ITDs. VAF is calculated by AO/DP, where AO, the abbreviation for Alternate allele Observation count, is the number of ITD supporting reads and DP is the total read depth. AO is counted from both ITD containing chimeric reads and split reads clipped at the same genomic location with chimeric reads. As showed in **Figure 1C**, the soft-clipped part of ITD containing read is used as seed sequence and each mismatched alignment of 3' or 5' read ends flanking the ITD will be aligned in pairwise with the seed using Smith-Waterman algorithm to add them in support of the ITD. With this procedure, soft-clipped reads result from ITD but were not recognized as ITD containing reads in step 1.3.b will be rescued in AO estimation to accurately measure the ITD allele frequency. Finally, the predicted ITDs will be reported in VCF format.

String rotation algorithm to determine duplications from reads with insertions

For the redefined CIGAR string with an inserted sequence in between two mapped parts, we used a string rotation method to judge whether the inserted sequence is a duplicated genomic sequence or a novel sequence. The duplication event is inferred based genomic sequence surrounding the insertion as described below and **Supplementary Figure S1**.

Algorithm 1 duplication inference from an inserted sequence

Require: Seq_{INS}, Len_{INS}, Seq_L, Seq_R

Seq_{INS} – Inserted sequence

Len_{INS} – The length of the inserted sequence

Seq_L, Seq_R – The left-side and the right-side genomic sequences adjacent to the inserted sequence from the reference genome, their length is 1bp less than the inserted sequence.

insertionInspector(Seq_{INS}, Len_{INS}, Seq_L, Seq_R)

1. **for** i = 1 **to** $Len_{INS}/2$ **do** /* left rotation */
2. $Seq_{INS} \leftarrow Len_{INS}^{th}$ element of Seq_{INS} + first Len_{INS} -1 elements of Seq_{INS}
3. $Seq_{EXTRACT} \leftarrow$ last i element of Seq_L + first $Len_{INS} - i$ elements of Seq_R

```
    if countMismatches(Seq_INS, Seq_EXTRACT) < cutoff then
        return true
    end if
end for
for i = 1 to Len_INS/2 do    /* right rotation */
    Seq_INS ← first Len_INS -1 elements of Seq_INS + 1^th element of Seq_INS
    Seq_EXTRACT ← last Len_INS – i elements of Seq_L + first i elements of Seq_R
    if countMismatches(Seq_INS, Seq_EXTRACT) < cutoff then
        return true
    end if
end for
return false
end
```

Simulated ITD dataset for FLT3 region

The simulated data were generated by ITDsim **[11]** that targeting FLT3-ITD hotspot region chr13:28,607,161–28,609,590 (hg19). The data set included a total of 40,401 samples with combinations of varied ITD lengths (range: 1–201 bp; n = 201) and varied starting positions (chr13:28,608,112–28,608,312; n = 201). ITD allele frequency was defined as 50% with the mixture of 1000 paired-end ITD reads and 1000 paired-end wild-type reads of varied read length (2x100bp and 2x275bp).

Simulated genome-wide ITD dataset

In order to evaluate ScanITD and make a comparison with other widely used duplication detection methods, we rearranged human chromosome 20 (GRCh37/hg19) using the program RSVSim [12] and svsim [13]. In total, we simulated 1,000 tandem duplications with the size ranging from 3bp to 300bp following a beta distribution to reflect the typical ITD size range [5] and real variant size distribution based on an estimation from the Database of Genomic Variants (DGV) [14]. Since ITDs reside within coding exons, we restrict the simulation to coding regions according to the UCSC Genome Browser RefSeq track file.

Based on the rearranged genome and unarranged genome, dwgsim [15] was used to generated synthetic sequence data for use as tumor samples. We generated 36 sets of paired-end reads with varying properties: A mean insert size of 500bp with 50bp standard deviation and 75, 100, 150 and 200 bp read lengths at 20×, 50× and 100× sequence depth each with 10%, 20% and 50% VAF. Detailed simulation procedure description and coding scripts are included in the Supplementary Methods.

Evaluation metrics for duplication calls

For simulated ITD dataset against *FLT3* gene region, we considered the predicted duplication (DUP) calls for each evaluated algorithm to be true positives (TP) if the prediction met the following criteria: (i) the predicted left breakpoint was within the FLT3-ITD hotspot region; (ii) the predicted size is equal to the true size. For simulated genome-wide ITD dataset, we used stringent criteria for the TP definition: (i) the predicted left breakpoint was within 1 bp of the true breakpoint; (ii) the predicted size is

equal to the true size. ITDs could also be detected as short insertions by some algorithms, we counted them as predicted DUP calls. False positives (FP) are predictions not satisfying the criteria. False negatives (FN) are DUP events not identified by the detection algorithm. To assess the performance of each tool, we used precision (or positive predictive value), recall (or sensitivity) and F1 score as evaluation metrics as defined below:

$$precision = \frac{TP}{TP + FP}$$

$$recall = \frac{TP}{TP + FN}$$

$$F1\ score = \frac{2TP}{2TP + FP + FN}$$

Reference duplication dataset for NA12878 data

A reference DUP dataset corresponding to NA12878 was generated by combing the DUP data identified from the NA12878 assembly generated with long reads (PacBio and ONT) using Sniffles[16] and the DUP data identified from the NA12878 assembly generated with Illumina short reads using Delly [17], Lumpy [18] and Manta [19]. These datasets are available at [20]. The merge of the DUP datasets was conducted using SURVIVOR[21], after the selection of DUP length ≥ 50bp, resulting in 1,560 DUPs totally.

Algorithms evaluation for NA12878 data

NA12878 WGS raw fastq files were obtained from European Nucleotide Archives (Accession number: ERR194147). Paired-end reads were aligned to the GRCh37 human reference using BWA-MEM v0.7.12 with default parameters and duplicated reads were discarded using Picard MarkDuplicates v1.68 [22]. Pindel (v0.2.5) [8], SoftSV (v1.4.2) [23], SvABA (v1.1.3) [24], ScanITD and Whamg (v1.7.0) [25] were employed for DUP calling for NA12878. We excluded ITDSeek, Genomon-ITDetector that did not work in our computational environment, and Delly that was used to generate the reference DUP call set.

To reduce confounding effects of detection strategies and differing conventions implemented by the different SV algorithms, we allowed some differences between breakpoint locations for different algorithms when comparing overlaps between DUP callsets with the reference one. Up to 20 bp of difference in the left breakpoints is allowed, 90% overlapped with the reference DUP, and the right breakpoint should not excess 20bp of the reference DUP. For tools reporting AO, DP and AF, such as Pindel and ScanITD, AO≥3, DP≥10 and AF≥0.01 were used as the threshold cutoffs. For SoftSV, the number of supporting reads≥3 as the cutoff. We extracted the predicted DUPs (≥ 50bp) from all tools we used and compared them against the reference DUP callset from NA12878 to measure the precision and recall of each method.

Results

Evaluation of ITD detection algorithms using simulated FLT3-ITD data

As ITDs most frequently occur in *FLT3* gene of AML patients, we first sought to compare ScanITD with three existing ITD detectors: ITDseek v1.2 [11], Genomon-ITDetector [26] and Pindel v0.2.5 [8] using two simulation datasets of hotspot FLT3-ITDs at 275 bp and 100 bp paired-end reads. ITDseek and Genomon-ITDetector are designed for ITD detection. It was reported Pindel also had a good performance in FLT3-ITD detection[2], so it was included in the comparison. We excluded ITD assembler [27] as it did not work in our computational environment and lacked support from its authors. The FASTQ files with synthetic paired-end reads were aligned by BWA-MEM to obtain BAM files. The BAM files with hard-clipped/soft-clipped reads or reads with small insertions were kept. All the evaluating ITD detectors were called using default parameters with minor adjustments and the analyses were based on used BWA-MEM aligned BAM files.

We observed that ScanITD achieved the highest recall, precision and F1 score under these two different reads length scenarios (Figure 2A). When further evaluating the recall and precision in different ITD size range, we found ScanITD is superior to detect medium to large size ITDs (>100bp) comparing with other methods (Figure 2B).

Evaluation of ITD detection algorithms using simulated genome-wide ITD dataset

To evaluate the performance of ITD detection algorithms in general, we generated a genome-wide ITD simulation dataset allowing unbiased estimation of the sensitivity and specificity of different ITD detection algorithms in other gene regions. To keep a reasonable runtime, we rearranged the target genome sequence based on human chromosome 20, which accounts for 2% of the human genome but has reasonably representative genomic features such as GC content, gene density and repeat content, compared with the whole genome. Then, we randomly placed 1,000 tandem duplications throughout the target genome. The size of the duplications ranged from 3bp to 300bp. To assess the impact of sequencing properties (i.e. reads depth and reads length) and duplication property (i.e. VAF), we generated 36 sets of synthetic paired-end reads with varied reads depth, reads length and VAF for the tandem duplications. Lastly, the simulation data were aligned to the human reference genome (GRCh37/hg19) using BWA-MEM.

Algorithms that developed for general use to identify SVs could detect tandem duplications at a larger scale. Therefore, we expanded our comparison to include several widely used SV detection tools. Besides the three ITD detectors (ITDseek, Genomon-ITDetector and Pindel), we compared ScanITD with four SV detectors including Delly v0.8.2, SvABA v1.1.3, SoftSV v1.4.2 and Whamg v1.7.0 on the simulated genome-wide ITD datasets with various reads depth, reads length and VAF settings. The measurement metrics precision (or positive predictive value), recall (or sensitivity) and F1 score (an overall measure of accuracy that combines precision and recall) were used to assess the performance of different algorithms in the comparison.

At the 10% VAF setting, we observed that Delly and Genomon-ITDetector achieved the highest precision across all coverage levels and reads lengths, with a poor performance in recall (Figure 3). ScanITD got the second runner-up position when reads length was 75bp or sequencing depth was 100×, suggesting it reliably detected tandem duplications in the short-read scenario, especially for the targeted sequencing setting. In terms of sensitivity, Pindel had the highest recall at a cost of low precision, followed by ScanITD in all tested situations at the 10% VAF setting. The differences were negligible at 50/100× coverage with 150/200 bp read lengths. While at 20% and 50% VAF settings, ScanITD showed the same or higher recall compared to Pindel at 50/100× coverage with 150/200 bp reads length (Supplementary Figure S2-S3). When considering both precision and recall, ScanITD achieved the highest F1 score of all the methods tested in all tested situations (Figure 3, Supplementary Figure S2-S3), indicating it could correctly identify real tandem duplications without being disturbed by false negatives. In general, our results showed that ScanITD had the best overall performance measured by F1-score in detecting tandem duplication events across all conditions.

We next sought to evaluate the performance of the VAF estimation by ScanITD together with ITDseek and Pindel using our simulated data. We choose these two existing ITD detectors for comparison as they are the only ITD detectors with the feature of reporting the VAFs of their predicted ITDs. As showed in Figure 4, all three methods tended to have a lower estimation of VAFs comparing their ground truth values, which may be explained by the non-uniform distribution of reads coverage [28]. Among them, ScanITD and Pindel reported more accurate VAFs than ITDseek. In general, ScanITD outperformed Pindel with a relatively higher median VAF estimation at most of the VAF settings in either low or high coverage data sets.

Evaluation with NA12878 WGS data

To assess the performance of ScanITD with real DNA sequencing data, we analyzed a well-studied HapMap sample NA12878/HG001. The 100-bp paired-end WGS data with an average coverage of 50× was provided through Illumina's Platinum genomes project [29]. We constructed a reference call set for sample NA12878 by combining duplication events identified from long reads (PacBio and ONT) and Illumina short reads. The vast majority of the called duplications are longer than 50bp, so DUPs with the length ≥ 50bp were used in the reference call set.

As ITDseek and Genomon-ITDetector are not capable to detect any duplications in this NA12878 data set, we tested ScanITD along with Pindel, SvABA, SoftSV and Whamg against reference duplication call set by measuring their precisions, recalls and F1 scores. We observed that ScanITD achieved the highest F1 score and second-highest precision/sensitivity among these five algorithms (Figure 5A), suggesting an overall better accuracy of detecting the duplication events in NA12878. When further evaluating the performance in different duplication size ranges, we found ScanITD is superior to detect small to medium size duplications (50-300bp) comparing with other methods (Figure 5B). Our results indicated ScanITD is the best approach to detect ITD range duplication events (<=300bp).

Finally, we recorded the computational runtime and memory consumption of ScanITD when analyzing the NA12878 WGS data set using a server equipped with a 16-core Intel Xeon(R) CPU E5-2620 v4 @ 2.10GHz with 16GB of memory. The first step (ITD reconstruction) spent 7 hours and the second step (split read realignment and ITD calling) spent 28 hours when running on chromosomes in parallel. Notably, ScanITD is memory-efficient by only requiring 2.2GB peak memory usage.

Application to whole-exome data of acute myeloid leukemia patients

To examine whether ScanITD could enhance ITD detection in clinical data, we analyzed the WES data from the TCGA AML cohort. It has been reported that 24 patients harbor experimentally validated *FLT3* ITDs [1]. We applied ScanITD together with the three existing ITD detectors to identify *FLT3* ITDs from these 24 patients. We used the original reported ITD size (ranging from 18bp to 102bp) as the gold standard [1] to measure the correctness of ITD prediction for each tool. As showed in Table 1, ScanITD correctly identified 22 ITDs and it demonstrated the highest sensitivity (92%) comparing with Pindel (72%), ITDseek (42%) and Genomon ITDetector (71%). We further evaluated the reported VAFs for the ScanITD, Pindel and ITDseek which are capable of predicting ITD allele fraction. We found ScanITD reported the highest VAFs in 20 samples while Pindel only reported the highest VAFs in 4 samples, and ITDseek always reported lower VAFs than ScanITD and Pindel. Thanks to split read local realignment, ScanITD could rescue ITD supporting reads in the AO calculation, increasing the reported VAF. For example, ScanITD reported an 87bp FLT3-ITD with its VAF of 0.30 that is nearly twice of the VAF reported by Pindel (0.16) in patient TCGA-AB-2844. A manual review of the aligned reads in this patient further confirmed ScanITD's estimation of this VAF was accurate (Supplementary Figure S4). Our results suggest that ScanITD outperforms the existing methods for accurately measuring the VAFs of the ITD predictions.

Discussion

Herein, we have devised ScanITD, a computational approach allowing the accurate identification of ITDs from DNA-Seq data. ScanITD made good use of chimeric alignments for ITDs reconstruction. By performing local realignment of clustered split reads, ScanITD achieved robust variant allele frequency estimation. The evaluations on simulated and real data demonstrate that ScanITD outperformed the existing ITD detectors, especially for estimating VAF with high accuracy. Compared to general-purpose SV detectors, ScanITD also exhibited competitive performance and superior accuracy in duplication detection, especially for ITD size ranges.

Besides performance improvements compared to other ITD callers, one improvement of ScanITD is the ability to distinguish insertions of novel sequence and insertions as a result of the duplicated genome sequence. Most of the ITD callers and general-purpose SV detection methods, such as Pindel [8] and SvABA [24], are not able to differentiate small-sized novel sequence insertions with tandem duplications and report both types of events as insertions. In essence, ScanITD belongs to split-read based approaches leveraging the split reads that solely mapped around ITD breakpoints. Other split-read based methods, such as Pindel and SoftSV[23], rely on realigning all the split reads.

Another novel feature of ScanITD is that it realigns split reads with a heuristic manner that can utilize all related split reads without limiting the length of the soft-clipped part. However, Pindel and SoftSV realign split reads to reference genome by requiring the soft-clipped part to be in a reasonable length (e.g. >10bp). The heuristic algorithm used by ScanITD is a seed-and-realignment procedure. Once the seed chimeric read was found, any split reads clipped at the same genomic location with the seed will be taken into consideration, no matter how short they were (Figure 1C and Supplementary Figure S4). This strategy makes ScanITD estimating VAFs with high accuracy (Figure 4 and Table 1). Our benchmarks using simulated data have demonstrated that ScanITD exhibited competitive or superior performance with algorithms using a split-read realignment strategy (e.g., Pindel, SoftSV) or integrated strategy (e.g., Delly integrating split-read and read-pair information) (Figure 3).

As a heuristic algorithm, ScanITD utilizes chimeric reads or reads with small insertions to locate the ITD breakpoints, the generation of these ITD indicators solely relies on soft-clipping aware NGS aligners such as BWA-MEM. However, under certain circumstances depending on ITD length and reads length, aligners may generate only soft-clipped/hard-clipped reads instead of chimeric reads or reads with small insertions. Under these cases, ScanITD is not able to determine correct breakpoints. (Figure 2B).

There are still limitations for ScanITD to detect some types of duplication events. Our benchmarks using NA12878 WGS data demonstrated that ScanITD showed a weak performance compared with general-purpose SV detection algorithms for large-size duplications. The existence of duplications carrying indels and dispersed duplication might be two possible reasons (Supplementary Figure S5). Under these cases, ScanITD is not able to determine correct breakpoints using chimeric reads. A combination and integration of multiple independent evidence such as read-pair and read depth information may further improve ScanITD's performance for non-tandem duplication event detection.

Conclusions

We presented ScanITD as a robust method for detecting ITDs from NGS data and predict precise ITD allele fraction. We demonstrated that ScanITD reliably detects medium and large size ITDs with synthetic and real data and outperformed the existing methods. ScanITD is capable of detecting ITD across the full-size spectrum with base-pair resolution. We anticipate ScanITD will enable identification and elucidation of clinically important ITDs that are currently difficult to characterize.

Availability of supporting source code and requirements

Project name: ScanITD
Project home page: https://github.com/ylab-hi/ScanITD
Operating system(s): platform independent
Programming language: Python
Other requirements: SAMTools (https://www.htslib.org/)

License: MIT License
Biotools identifier: ScanITD (https://bio.tools/ScanITD)
RRID: SCR_018886

Availability of supporting data and materials

Whole exome sequencing data from the TCGA AML cohort is available at the Genomic Data Commons Data Portal [30] (Project ID: TCGA-LAML; dbGaP study accession: phs000178). NA12878 WGS fastq data is available at European Nucleotide Archives (accession number: ERR194147). An archival copy of the code and supporting data is available via the *GigaScience* GigaDB database [31].

Declarations

List of abbreviations

AO: Alternate allele Observation count

AML: Acute Myeloid Leukemia

BAM: Binary Alignment Map

DP: Read Depth

ELN: European Leukemia Net

TDUP: Tandem Duplication

ITD: Internal tandem duplications

NGS: Next-Generation Sequencing

SV: Structural Variations

TCGA: The Cancer Genome Atlas

VAF: Variant Allele Frequency

WGS: Whole Genome Sequencing

WES: Whole Exome Sequencing

Ethics approval and consent to participate

Not applicable

Consent for publication

Not applicable

Competing interests

The authors declare that they have no competing interests.

Funding

This work was supported by Research Starter Grant from PhRMA foundation, Young Investigator Award from the Prostate Cancer Foundation and Idea Development Award from Department of Defense Prostate Cancer Research Program (W81XWH-19-1-0161). The work is also supported by The Eagles Telethon Post Doctoral Fellowship to T.-Y. W.

Authors' contributions

T.-Y. W. developed the software, performed data analysis, and wrote the manuscript. R.Y. conceptualized the research idea, supervised the development of the software and data analysis, and reviewed and edited the draft.

Acknowledgements

We thank Dr. Jeffrey McDonald, Tim Lastine and Theresa Tucker at The Hormel Institute for their technical support for computing facilities. Support from the Minnesota Supercomputer Institute (MSI) is also gratefully acknowledged.

References

1. Cancer Genome Atlas Research N, Ley TJ, Miller C, Ding L, Raphael BJ, Mungall AJ, Robertson A, Hoadley K, Triche TJ, Jr., Laird PW, et al: **Genomic and epigenomic landscapes of adult de novo acute myeloid leukemia.** *N Engl J Med* 2013, **368:**2059-2074.
2. Spencer DH, Abel HJ, Lockwood CM, Payton JE, Szankasi P, Kelley TW, Kulkarni S, Pfeifer JD, Duncavage EJ: **Detection of FLT3 internal tandem duplication in targeted, short-read-length, next-generation sequencing data.** *J Mol Diagn* 2013, **15:**81-93.
3. Wertheim GB, Daber R, Bagg A: **Molecular diagnostics of acute myeloid leukemia: it's a (next) generational thing.** *J Mol Diagn* 2013, **15:**27-30.
4. Griffith J, Black J, Faerman C, Swenson L, Wynn M, Lu F, Lippke J, Saxena K: **The structural basis for autoinhibition of FLT3 by the juxtamembrane domain.** *Mol Cell* 2004, **13:**169-178.
5. Wang X, Yan Z, Fang P, Liu W, Glynn S, Biroschak J, Galderisi C, Spittle C, Li J: **Abstract 4522: An optimized NGS workflow for detection of FLT3 internal tandem duplication (ITD) in AML samples.** *Cancer Research* 2018, **78:**4522.
6. Daver N, Schlenk RF, Russell NH, Levis MJ: **Targeting FLT3 mutations in AML: review of current knowledge and evidence.** *Leukemia* 2019, **33:**299-312.
7. Ding L, Ley TJ, Larson DE, Miller CA, Koboldt DC, Welch JS, Ritchey JK, Young MA, Lamprecht T, McLellan MD, et al: **Clonal evolution in relapsed acute myeloid leukaemia revealed by whole-genome sequencing.** *Nature* 2012, **481:**506-510.
8. Ye K, Schulz MH, Long Q, Apweiler R, Ning Z: **Pindel: a pattern growth approach to detect break points of large deletions and medium sized insertions from paired-end short reads.** *Bioinformatics* 2009, **25:**2865-2871.
9. Li H: **Aligning sequence reads, clone sequences and assembly contigs with BWA-MEM.** In *ArXiv e-prints*. pp. 2013:arXiv:1303.3997; 2013:2013:arXiv:1303.3997.
10. Yang R, Van Etten JL, Dehm SM: **Indel detection from DNA and RNA sequencing data with transIndel.** *BMC Genomics* 2018, **19:**270.

11. Au CH, Wa A, Ho DN, Chan TL, Ma ES: **Clinical evaluation of panel testing by next-generation sequencing (NGS) for gene mutations in myeloid neoplasms.** *Diagn Pathol* 2016, **11:**11.
12. Bartenhagen C, Dugas M: **RSVSim: an R/Bioconductor package for the simulation of structural variations.** *Bioinformatics* 2013, **29:**1679-1681.
13. svsim Github: **https://github.com/mfranberg/svsim.** Accessed on 6th January 2020.
14. Iafrate AJ, Feuk L, Rivera MN, Listewnik ML, Donahoe PK, Qi Y, Scherer SW, Lee C: **Detection of large-scale variation in the human genome.** *Nat Genet* 2004, **36:**949-951.
15. DWGSIM Github: **https://github.com/nh13/DWGSIM.** Accessed on 6th January 2020.
16. Sedlazeck FJ, Rescheneder P, Smolka M, Fang H, Nattestad M, von Haeseler A, Schatz MC: **Accurate detection of complex structural variations using single-molecule sequencing.** *Nat Methods* 2018, **15:**461-468.
17. Rausch T, Zichner T, Schlattl A, Stutz AM, Benes V, Korbel JO: **DELLY: structural variant discovery by integrated paired-end and split-read analysis.** *Bioinformatics* 2012, **28:**i333-i339.
18. Layer RM, Chiang C, Quinlan AR, Hall IM: **LUMPY: a probabilistic framework for structural variant discovery.** *Genome Biol* 2014, **15:**R84.
19. Chen X, Schulz-Trieglaff O, Shaw R, Barnes B, Schlesinger F, Kallberg M, Cox AJ, Kruglyak S, Saunders CT: **Manta: rapid detection of structural variants and indels for germline and cancer sequencing applications.** *Bioinformatics* 2016, **32:**1220-1222.
20. Structrual variation calls from NA12878 using long reads and short reads: **http://labshare.cshl.edu/shares/schatzlab/www-data/fsedlaze/Sniffles/NA12878/.** Accessed on 6th January 2020.
21. Jeffares DC, Jolly C, Hoti M, Speed D, Shaw L, Rallis C, Balloux F, Dessimoz C, Bahler J, Sedlazeck FJ: **Transient structural variations have strong effects on quantitative traits and reproductive isolation in fission yeast.** *Nat Commun* 2017, **8:**14061.
22. Picard website: **http://broadinstitute.github.io/picard/.** Accessed on 6th January 2020.
23. Bartenhagen C, Dugas M: **Robust and exact structural variation detection with paired-end and soft-clipped alignments: SoftSV compared with eight algorithms.** *Brief Bioinform* 2016, **17:**51-62.
24. Wala JA, Bandopadhayay P, Greenwald NF, O'Rourke R, Sharpe T, Stewart C, Schumacher S, Li Y, Weischenfeldt J, Yao X, et al: **SvABA: genome-wide detection of structural variants and indels by local assembly.** *Genome Res* 2018, **28:**581-591.
25. Kronenberg ZN, Osborne EJ, Cone KR, Kennedy BJ, Domyan ET, Shapiro MD, Elde NC, Yandell M: **Wham: Identifying Structural Variants of Biological Consequence.** *PLoS Comput Biol* 2015, **11:**e1004572.
26. Chiba K, Shiraishi Y, Nagata Y, Yoshida K, Imoto S, Ogawa S, Miyano S: **Genomon ITDetector: a tool for somatic internal tandem duplication detection from cancer genome sequencing data.** *Bioinformatics* 2015, **31:**116-118.
27. Rustagi N, Hampton OA, Li J, Xi L, Gibbs RA, Plon SE, Kimmel M, Wheeler DA: **ITD assembler: an algorithm for internal tandem duplication discovery from short-read sequencing data.** *BMC Bioinformatics* 2016, **17:**188.
28. Sims D, Sudbery I, Ilott NE, Heger A, Ponting CP: **Sequencing depth and coverage: key considerations in genomic analyses.** *Nat Rev Genet* 2014, **15:**121-132.
29. Eberle MA, Fritzilas E, Krusche P, Kallberg M, Moore BL, Bekritsky MA, Iqbal Z, Chuang HY, Humphray SJ, Halpern AL, et al: **A reference data set of 5.4 million phased human variants validated by genetic inheritance from sequencing a three-generation 17-member pedigree.** *Genome Res* 2017, **27:**157-164.

30. Genomic Data Commons Data Portal: **https://portal.gdc.cancer.gov/.** Accessed on 6th January 2020.
31. Wang T, Yang R: **Supporting data for "ScanITD: detecting internal tandem duplication with robust variant allele frequency estimation".** *GigaScience Database* 2020, **http://dx.doi.org/10.5524/100775**.

Tables

Table 1. FLT3-ITD detection in TCGA AML samples

TCGA sample ID	ITD (length)	ScanITD (VAF)	ScanITD (length)	Pindel (VAF)	Pindel (length)	ITDseek (VAF)	ITDseek (length)	Genomon ITDetector (length)
TCGA-AB-2812	51	**0.34**	51	0.14	51	0.11	51	51
TCGA-AB-2825	102	0.06	102	**0.17**	102	0.11	97	missed
TCGA-AB-2830	69	0.01	69	**0.027**	69	0.03	56	42
TCGA-AB-2836	33	**0.08**	33	0.047	33	0.03	34	missed
TCGA-AB-2840	18	**0.25**	18	0.23	18	0.01	18	18
TCGA-AB-2844	87	**0.30**	87	0.16	87	0.05	89	missed
TCGA-AB-2853	18	0.21	18	**0.30**	18	0.08	18	18
TCGA-AB-2869	54	**0.21**	54	0.5	46	0.12	55	54
TCGA-AB-2871	63	**0.011**	63	0.0059	63	0.01	63	missed
TCGA-AB-2875	30	**0.25**	30	0.16	30	0.03	30	30
TCGA-AB-2877	18	**0.22**	18	0.20	18	0.02	18	18
TCGA-AB-2879	33	**0.32**	33	0.20	33	0.06	34	33
TCGA-AB-2880	21	**0.23**	21	0.18	21	0.11	21	21
TCGA-AB-2895	45	**0.23**	45	0.16	45	0.09	49	45
TCGA-AB-2913	66	**0.14**	66	0.065	66	0.06	68	66
TCGA-AB-2915	51	**0.029**	51	0.13	54	0.03	51	51
TCGA-AB-2918	21	0.1	90	0.11	88	0.05	90	88
TCGA-AB-2921	24	0.15	57	0.09	57	0.06	53	24
TCGA-AB-2922	33	**0.25**	33	0.15	33	0.04	33	33
TCGA-AB-2925	42	**0.21**	42	0.10	42	0.11	45	42
TCGA-AB-2930	42	**0.05**	42	0.029	42	0.02	39	42
TCGA-AB-2931	75	**0.15**	75	0.28	70	0.07	72	missed
TCGA-AB-2934	57	**0.05**	57	0.043	57	0.05	56	57
TCGA-AB-2942	24	**0.15**	24	0.12	24	0.03	24	24

The correct predictions with the highest VAF are highlighted in bold, incorrect predictions are colored with red.

Figure Legends

Figure 1. ITD detection with ScanITD. A) Schematic overview of ScanITD; B) ITDs are recovered from primary (softclipping mode SM) and alternative alignments (softclipping mode MS) of chimeric reads (ITD size >= read length); C) Split read local realignment to calculate the actual number of ITD supporting reads (labeled by AO) that is the sum of ITD containing seed read and split reads clipped at the same genomic location with seed reads.

Figure 2. Benchmarking of ScanITD for ITD detection against existing ITD detection tools using 2×100bp and 2×275bp simulated reads. (A) Performance measured by precision (left panel), recall (middle panel) and F1 score (right) for ScanITD, ITDseek, Genomon-ITDetector, and Pindel. (B) Performance measured by precision (left panel) and recall (right panel) at ITD length range from 1bp to 201bp.

Figure 3. Benchmarking of ScanITD for ITD detection against existing ITD detection tools and SV detection tools using genome-wide simulated reads with 10% VAF.

Figure 4. Benchmarking VAF of ScanITD for ITD detection against ITDseek and Pindel using genome-wide simulated reads.

Figure 5. Benchmarking of duplication detection using NA12878 whole genome sequencing data. (A) Overall performance comparison. (B) Performance comparison in different duplication size range. Precision, recall and F1 score are used as the performance measurements.

Figure 1

Click here to access/download;Figure;Figure1.eps

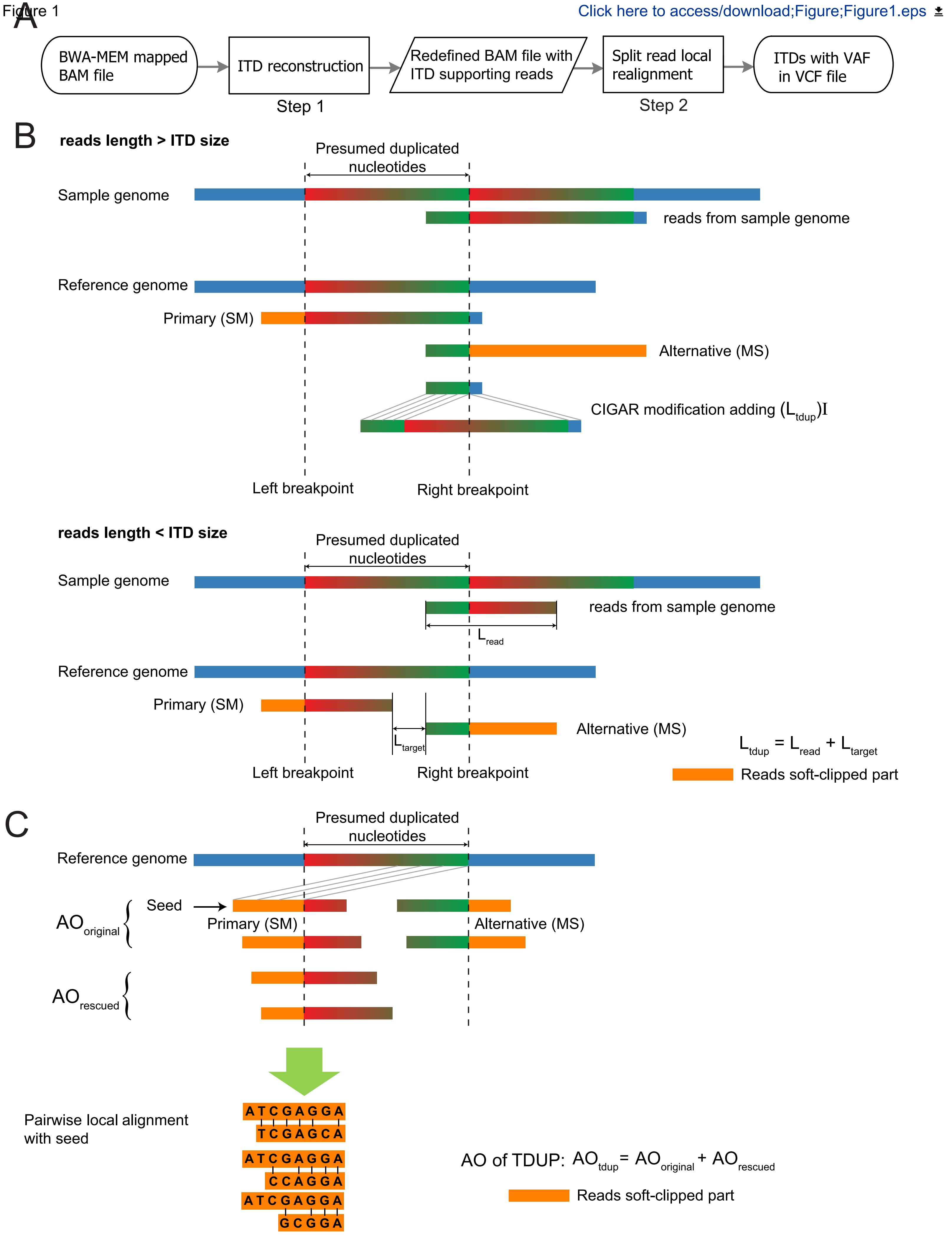

Figure 2

Click here to access/download;Figure;Figure2.eps

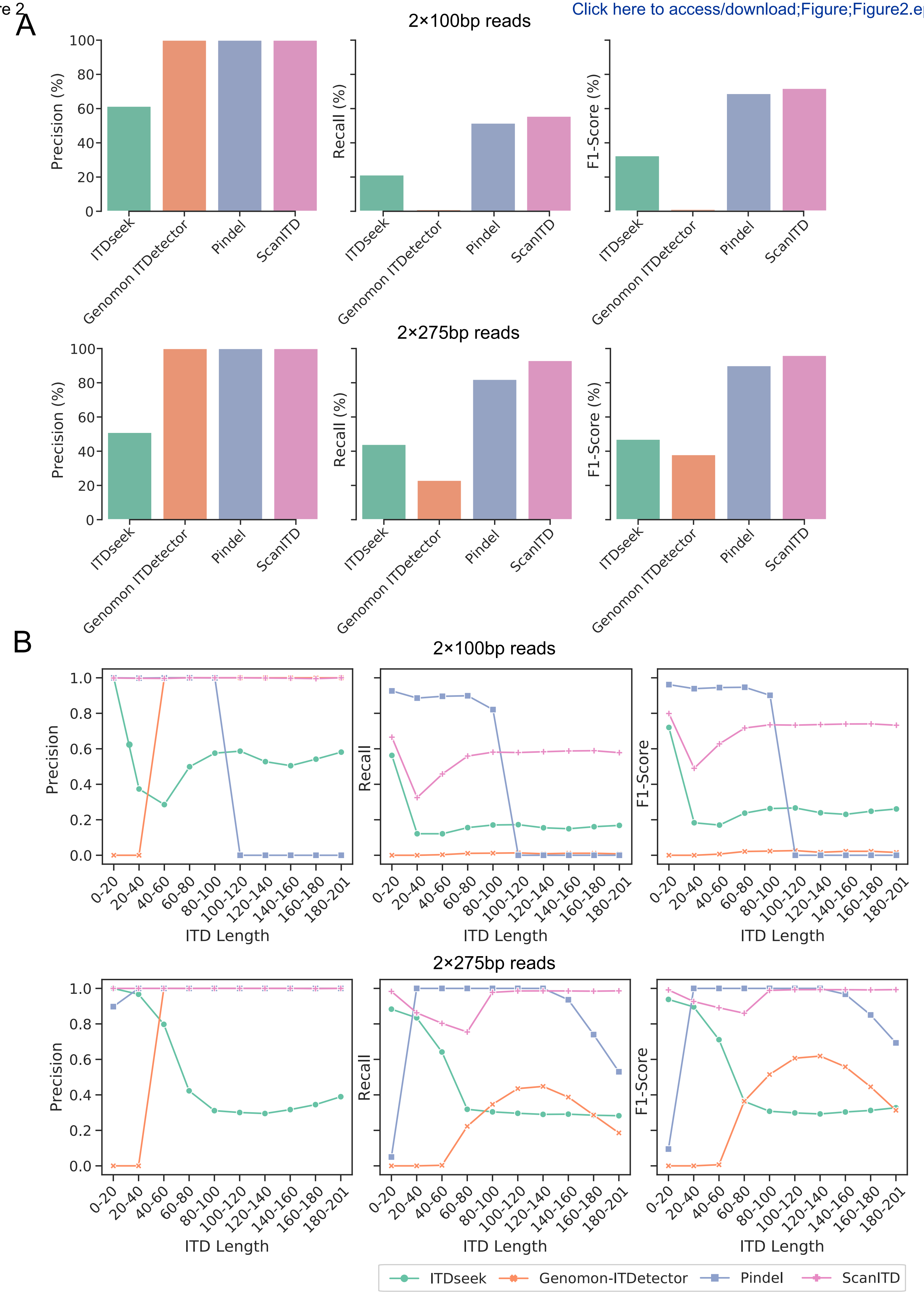

Figure 3

Click here to access/download;Figure;Figure3.eps

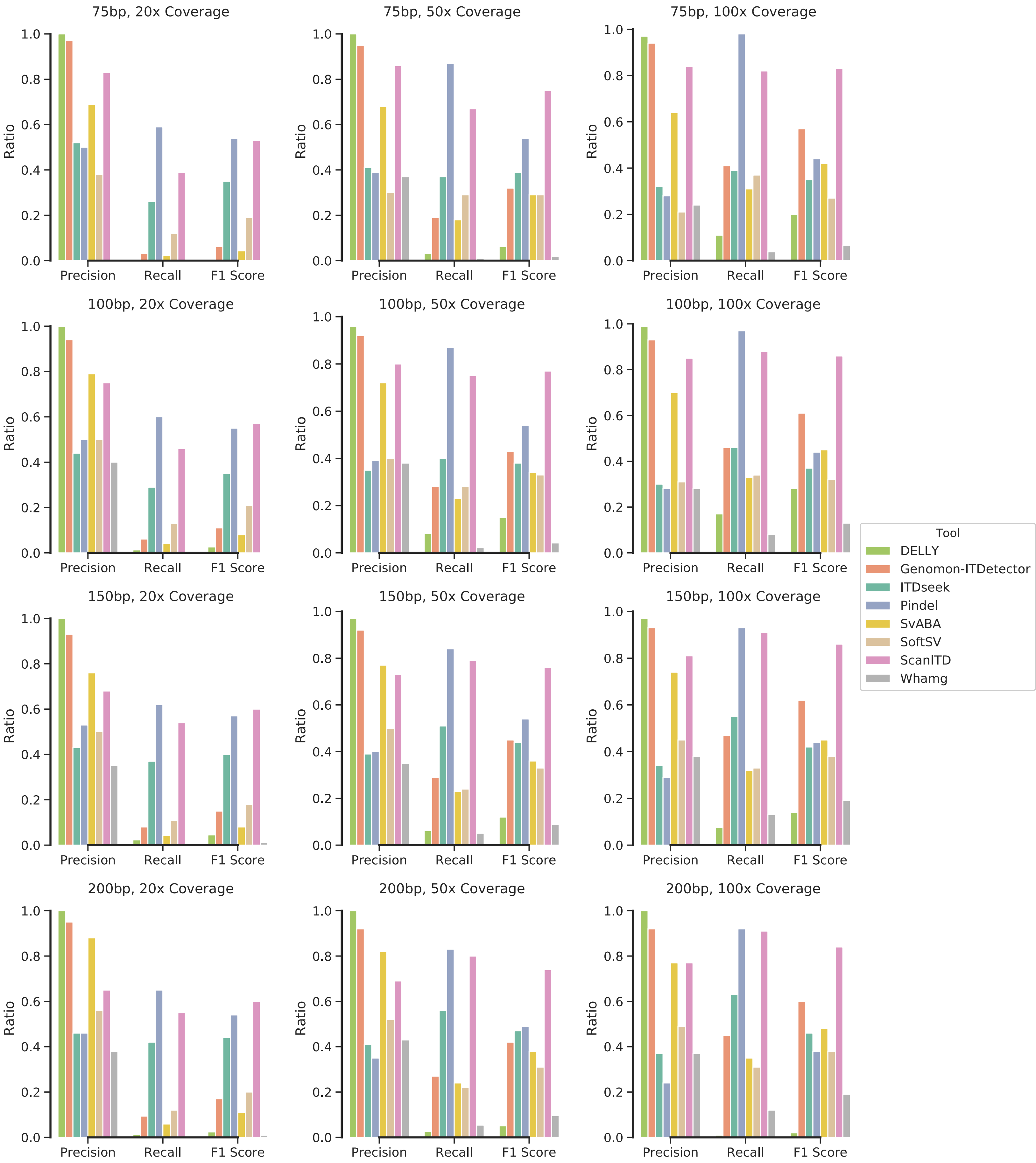

Figure 4 Click here to access/download;Figure;Figure4.eps

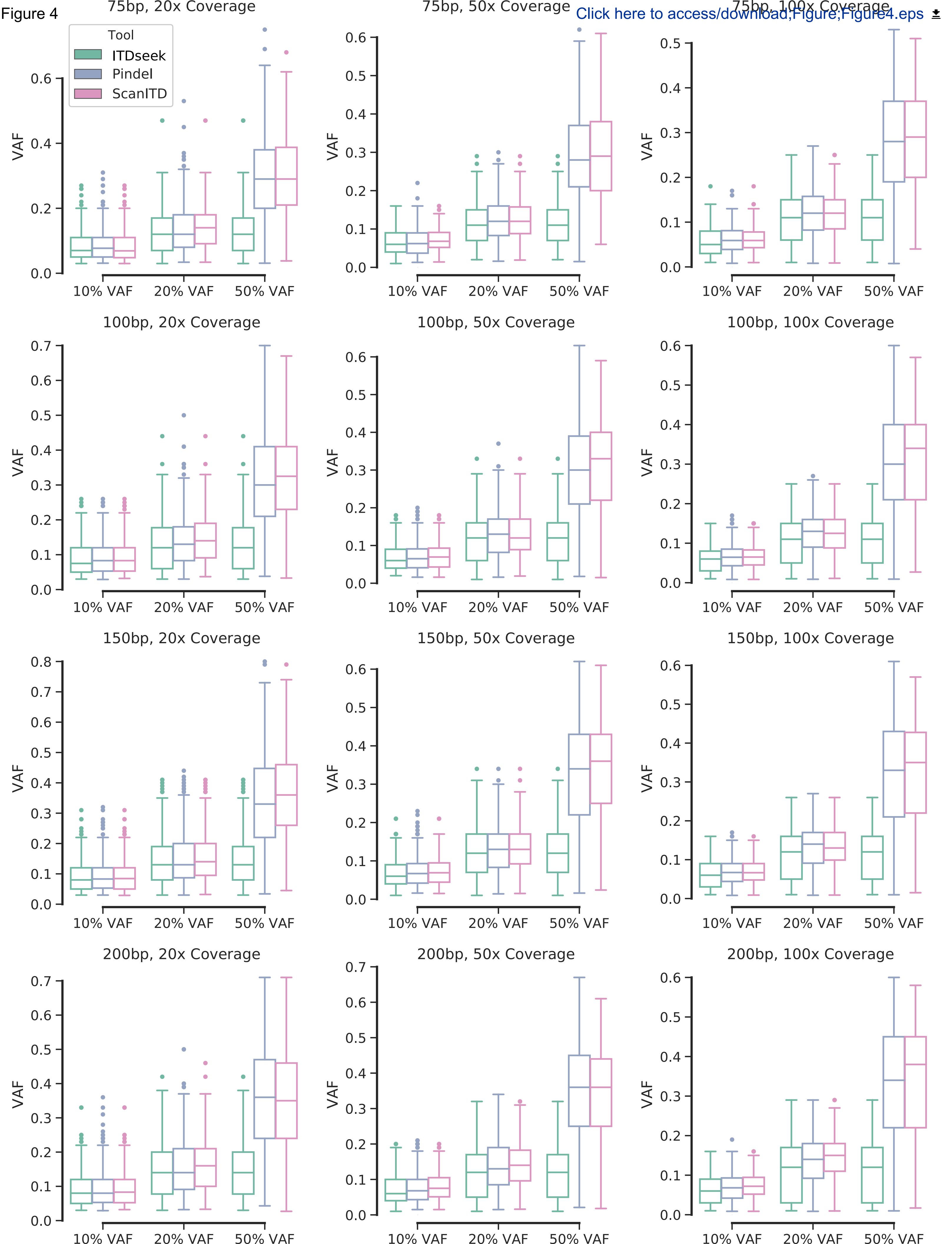

Figure 5

Click here to access/download;Figure;Figure5.eps

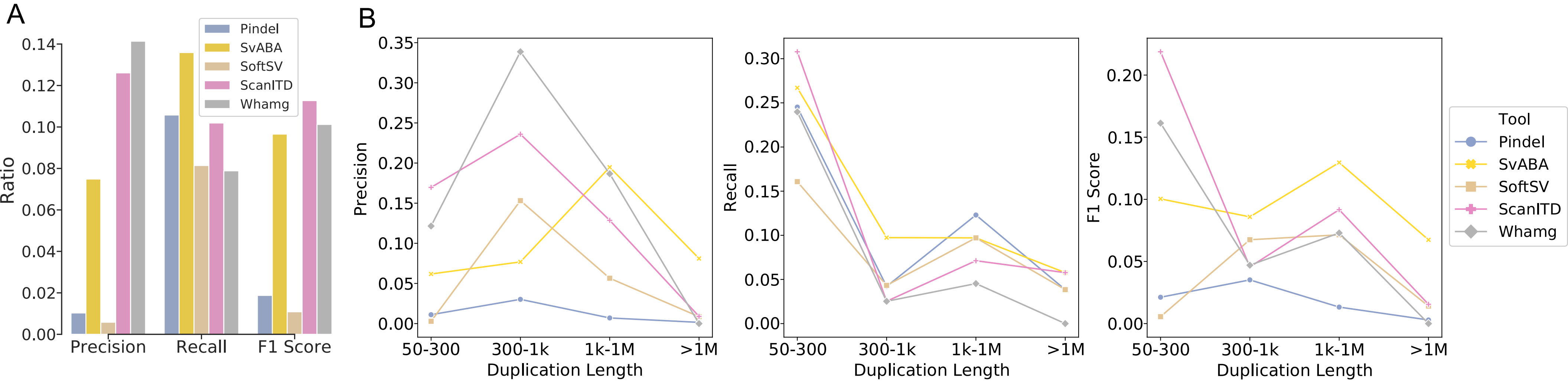

Click here to access/download
Supplementary Material
Supplementary_materials.docx
