# Supplementary material for: ScanITD: Detecting internal tandem duplication with robust variant allele frequency estimation
Source: Gigascience. 2020 Aug 27;9(8):giaa089. doi: 10.1093/gigascience/giaa089 (PMC7450668; doi:10.1093/gigascience/giaa089)
Supplement: giaa089_Supplemental_File [file giaa089_supplemental_file.docx]

*Supplementary Information*

**ScanITD: detecting internal tandem duplication with robust variant allele frequency estimation**

Ting-You Wang^1^ and Rendong Yang^1,2*^

^1^The Hormel Institute, University of Minnesota, Austin MN 55912

^2^Masonic Cancer Center, University of Minnesota, Minneapolis, MN 55455.

*To whom correspondence should be addressed (yang4414@umn.edu).

**Supplementary Methods**

**Simulated genome-wide ITD dataset**

The R package RSVSim (version 1.18.0)[1] was used to generate the positions of tandem duplications. We simulated 1,000 tandem duplications, the size ranges from 3bp to 300bp.

The main steps of preprocessing and call for RSVsim in R[2] are the following:

library(RSVSim)

sizeDups = estimateSVSizes(1000, minSize=3, maxSize=300, default="tandemDuplications",hist=FALSE)

# regions were generated using GRanges function with human CDS

# coordinate as input

sim = simulateSV(chrs='chr20', output=".", dups=1000, sizeDups=sizeDups, maxDups=2, regionsDups=regions, repeatBias=FALSE, seed=42, verbose=FALSE);

Svsim [3] was used to modified the human reference genome chr20 (GRCh37/hg19) with tandem duplication positions from RSVSim. The function call looks like:

python create_indel_genome.py chr20.hg19.fa tandemDuplications.txt chr20.hg19.TDUP.fa

Simulation of Illumina paired-end reads was done with dwgsim[4] for varying sequencing parameters:

(a) LENGTH was set to 75, 100, 150 and 200 (in bp).

(b) Insert size was set to 500bp and its standard deviation to 50.

(c) The base error rate increases linearly form 0.05% at the 5’ end to 0.3% at the 3’ end for each read.

(d) VAF for the tandem duplication was set to 10%, 20% and 50%.

(e) The targeting reads depths were 20**×**, 50**×** and 100**×**.

In order to obtain the synthetic reads for each combination of sequencing depth, VAF and reads length, we simulated reads using both the modified genome and the vanilla genome. Let take 100bp length reads, at sequencing depth of 100**×** with 20% VAF as an example. The function call looks like:

dwgsim -C 20 -1 100 -2 100 -d 500 -s 50 -c 0 -R 0 -e 0.0005-0.003 -E 0.0005-0.003 chr20.hg19.TDUP.fa mutated_reads

dwgsim -C 80 -1 100 -2 100 -d 500 -s 50 -c 0 -R 0 -e 0.0005-0.003 -E 0.0005-0.003 chr20.hg19.fa wildtype_reads

Then by concatenating the mutated reads and wild-type reads, the raw paired-end sequences were obtained.

**Sequence alignment and post-processing**

The paired-end reads (synthetic and real) were aligned with BWA-MEM[5], the alignment file was sorted by samtools [6], and duplicated reads were removed with Picard [7]:

(a) GENOME is the human reference genome (GRCh3/hg19) in FASTA format.

(b) FASTQ1, FASTQ2 are the raw paired-end sequences in FASTQ format.

The main steps of mapping and postprocessing are the following:

bwa mem -M -R $READS_GROUP $GENOME $FASTQ1 $FASTQ2 | samtools sort -O BAM -o $BWA_BAM –

picard MarkDuplicates I=$BWA_BAM O=$BAM REMOVE_DUPLICATES=true ASSUME_SORTED=true VALIDATION_STRINGENCY=LENIENT CREATE_INDEX=true

**References**

1. Bartenhagen C, Dugas M: **RSVSim: an R/Bioconductor package for the simulation of structural variations.** *Bioinformatics* 2013, **29:**1679-1681.

2. Team RC: **R: A Language and Environment for Statistical Computing.** 2017.

3. svsim GitHub: **https://github.com/mfranberg/svsim.** Accessed on 6th January 2020.

4. DWGSIM GitHub: **https://github.com/nh13/DWGSIM.** Accessed on 6th January 2020.

5. Li H: **Aligning sequence reads, clone sequences and assembly contigs with BWA-MEM.** In *arXiv e-prints*; 2013.

6. Li H, Handsaker B, Wysoker A, Fennell T, Ruan J, Homer N, Marth G, Abecasis G, Durbin R, Genome Project Data Processing S: **The Sequence Alignment/Map format and SAMtools.** *Bioinformatics* 2009, **25:**2078-2079.

7. Picard website: **http://broadinstitute.github.io/picard/.** Accessed on 6th January 2020.

**Supplementary Figures**

**Figure S1: A string rotation method to determine whether the inserted sequence is from a duplicated genomic sequence or not.**

**Figure S2: Benchmarking of ScanITD for ITD detection against existing ITD detection tools and SV detection tools using genome-wide simulated reads with 20% VAF.**

**Figure S3: Benchmarking of ScanITD for ITD detection against existing ITD detection tools and SV detection tools using genome-wide simulated reads with 50% VAF.** DELLY (v0.8.2) detected no duplications at 200bp, 50/100**×** coverage settings, which might be a bug in it.

**Figure S4: Experimentally validated *FLT3* ITD (chr13:28608215-28608301) was identified by ScanITD in TCGA-AB-2844 WES data.** AO is counted from both ITD containing chimeric reads and split reads clipped at the same genomic location with chimeric reads, resulting in an increased VAF. AO_original_=3, AO_rescued_=18, DP=70, VAF=0.3. The colored part in each read shows a soft-clipped fragment.

**Figure S5: Illustration of the non-tandem duplication scenarios with chimeric reads.** (A) duplication carrying an insertion. (B) dispersed duplication.
