# Supplementary material for: ScanITD: Detecting internal tandem duplication with robust variant allele frequency estimation
Source: Gigascience. 2020 Aug 27;9(8):giaa089. doi: 10.1093/gigascience/giaa089 (PMC7450668; doi:10.1093/gigascience/giaa089)
Supplement: giaa089_Response_to_Reviewer_Comments_Original_Submission [file giaa089_response_to_reviewer_comments_original_submission.pdf]

# Author's Response To Reviewer Comments

Close

Dr. Hans Zauner, PhD
Assistant Editor of GigaScience

July 28, 2020
RE: GigaScience - GIGA-D-20-00166

Dear Dr. Zauner,

We would like to thank you for accepting manuscript (GIGA-D-20-00166) entitled "ScanITD: detecting internal tandem duplication with robust variant allele frequency estimation" for publication in the journal of GigaScience. We greatly appreciate the critical examination and the constructive comments by the reviewers. The manuscript has been revised for better readability according to the suggestions of the Editor and Reviewers.

We already have submitted the supporting data to GigaDB (http://dx.doi.org/10.5524/100775). Following Editor's editorial requests, we added GigaDB citation, provided identifiers of bio.tools and SciCrunch.org, and moved the URLs in the main text to the bibliography. We have made all the necessary corrections.

We again appreciate the kindness of the Editor and Reviewers in helping improve the manuscript. Thank you again.

Best regards,

Rendong Yang, Ph.D.
Assistant Professor
The Hormel Institute
University of Minnesota
Tel: 507-437-9660
Email: yang4414@umn.edu

Close
